# Arrays of ultraconserved non-coding regions span the loci of key developmental genes in vertebrate genomes

**DOI:** 10.1186/1471-2164-5-99

**Published:** 2004-12-21

**Authors:** Albin Sandelin, Peter Bailey, Sara Bruce, Pär G Engström, Joanna M Klos, Wyeth W Wasserman, Johan Ericson, Boris Lenhard

**Affiliations:** 1 Center for Genomics and Bioinformatics, Karolinska Institutet, Stockholm, Sweden; 2 Department of Cell and Molecular Biology, Karolinska Institutet, Stockholm, Sweden; 3 Department of Biosciences at Novum, Karolinska Institutet, Stockholm, Sweden; 4 Centre for Molecular Medicine, Department of Medical Genetics, University of British Columbia, Vancouver, Canada

## Abstract

**Background:**

Evolutionarily conserved sequences within or adjoining orthologous genes often serve as critical *cis*-regulatory regions. Recent studies have identified long, non-coding genomic regions that are perfectly conserved between human and mouse, termed ultra-conserved regions (UCRs). Here, we focus on UCRs that cluster around genes involved in early vertebrate development; genes conserved over 450 million years of vertebrate evolution.

**Results:**

Based on a high resolution detection procedure, our UCR set enables novel insights into vertebrate genome organization and regulation of developmentally important genes. We find that the genomic positions of deeply conserved UCRs are strongly associated with the locations of genes encoding key regulators of development, with particularly strong positional correlation to transcription factor-encoding genes. Of particular importance is the observation that most UCRs are clustered into arrays that span hundreds of kilobases around their presumptive target genes. Such a hallmark signature is present around several uncharacterized human genes predicted to encode developmentally important DNA-binding proteins.

**Conclusion:**

The genomic organization of UCRs, combined with previous findings, suggests that UCRs act as essential long-range modulators of gene expression. The exceptional sequence conservation and clustered structure suggests that UCR-mediated molecular events involve greater complexity than traditional DNA binding by transcription factors. The high-resolution UCR collection presented here provides a wealth of target sequences for future experimental studies to determine the nature of the biochemical mechanisms involved in the preservation of arrays of nearly identical non-coding sequences over the course of vertebrate evolution.

## Background

Comparative genome sequence analysis, often termed phylogenetic footprinting, has proven successful for the identification of *cis*-regulatory regions[1,2]. Recent computational and experimental studies have identified a small number of large, highly conserved enhancers, or 'global control regions', associated with the regulation of important developmental genes such as *DACH *[3], *SOX9 *[4], *Dlx *bigene [5,6], and *HOX-D *[7,8] clusters. These regulatory regions can act at distances of several hundred kilobases from their target genes, while at the same time conferring an equivalent expression pattern to reporter genes over much shorter distances (e.g. [3]). A recent computational analysis proves that such highly conserved elements (termed ultra-conserved elements (UCRs)) are occurring far more often than expected [9]. In the study by Bejerano *et al*., UCRs are defined as regions perfectly conserved between human and mouse longer than 200 base pairs (bp). The study reports a significant association of a non-transcribed subset of those elements with DNA-binding proteins; an equivalent observation has been made independently by Boffeli *et al*.[10] for a limited number of most highly conserved elements between human and pufferfish. The stringent criteria for conservation applied in the two studies miss many known enhancer elements that are shorter than 200 bp, and highly conserved across all vertebrates. For instance, in a recently published study, Sabarinadh *et al*. [11] described a number of non-transcribed regions flanking the genes of *HoxD *gene cluster that are highly conserved across vertebrate genomes.

In this paper, we define a set of UCRs using high-resolution criteria that detect segments conserved between the human, mouse and pufferfish genomes. Analysis of this set provides insights into a previously unrealized organizational structure of UCRs in vertebrate genomes. We conclusively show that clusters of UCRs are globally associated with many of the genes that act as master regulators during vertebrate development. The clustered distribution of these regions along chromosomes and, importantly, around their presumptive target genes suggests that gene regulation involves the coordinated action of numerous, widely dispersed elements.

## Results

### Definition and genomic environment of ultra-conserved non-coding regions (UCRs)

We initiated this study by applying comparative genomics to identify putative regulatory regions for a number of evolutionary conserved homeodomain transcription factors that control neural cell fate determination [12,13]. When we examined the genomic landscapes surrounding homeodomain gene loci, we consistently found non-coding regions that exhibited a striking degree of sequence conservation between human and mouse over a minimum of 50 bp. Many of these regions are at least partially conserved over extended periods of evolution. The observed nucleotide identities between human and mouse sequences exceed even those of exon sequences encoding identical proteins. Such striking sequence conservation has previously been anecdotally associated with long-range enhancers for several developmental genes [3-8].

To test whether the association of UCRs with regulatory genes reflected a global genomic trend, we identified a comprehensive set of human/mouse/pufferfish UCRs for detailed analysis. We defined minimum requirements for a UCR (see Methods) and performed a genome-scale computational analysis that retrieved 3583 human/mouse/pufferfish UCRs. Since one of the requirements is that the UCRs are not overlapping actively transcribed genomic regions, they would belong to type II UCRs defined by Bejerano *et al*. [9].

The median UCR length was 125 bp, but extreme lengths (>1000 bp) were observed. Qualitative assessment of "genescapes", the gene structures, surrounding UCRs revealed them to be present either in introns, in dense clusters around a group of genes or in 'gene deserts' (up to several thousands kilobases from known genes). There appeared to be a strong association between locations of our set of UCRs and genes encoding transcription factors – even stronger than that reported by Bejerano *et al*.[9] [see Additional file 1 and 2]. This observation will be proven in the subsequent analysis.

### UCRs are strongly associated with DNA-binding proteins

**Figure 1:**
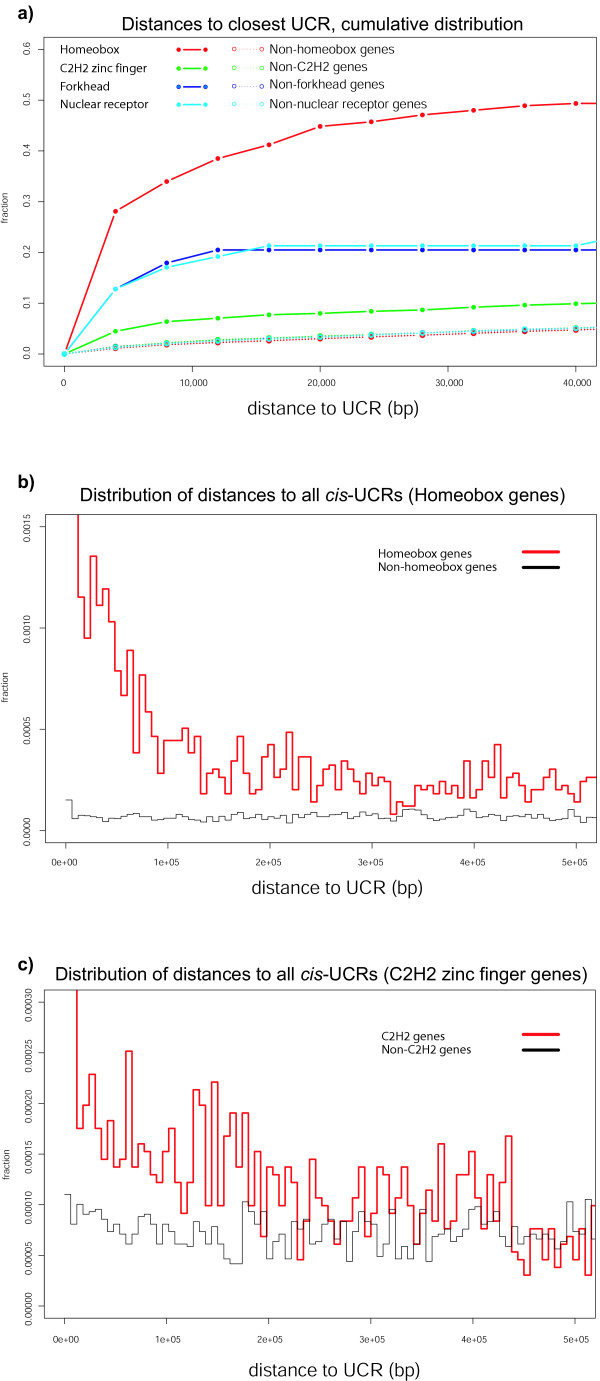
**Spatial correlation of transcription factor gene families to UCRs in the human genome**. **A**. Cumulative distribution of distances to the closest UCR for selected subsets of genes. Distance to the closer end of the transcript mapping (either 3' or 5'). Majority of major classes of transcription factors are closer to UCRs than random genes. **B, C**. Occurrence of UCRs around selected subsets of genes. This plot summarizes the distribution of distances to all UCRs on the same chromosome for each gene in the subset. There is a visible over-representation of UCRs up to 300 kb from homeobox genes, and up to 150 kb from C2H2 zinc finger genes.

To quantitatively assess the characteristics of genes proximal to UCRs, we analyzed the over-representation of gene annotations. We retrieved the InterPro [14] domain annotation for all genes adjacent to or containing UCRs. A statistical assessment (Fisher's exact test) of the observed domain biases for these genes was performed to assess the probability that the domain distributions were the same for the UCR genes as compared to the set of all genes. Even with a conservative (Bonferroni) correction for multiple testing [15], structural domains of transcription factors are significantly over-represented (P-value 9.33e-66) within the gene annotations (Table 1) [all domains are listed in Additional file 3 and 4]. In order to obtain robust results, we chose the four domains from Table 1 present in the highest number of proteins (homeobox, C2H2 zinc finger, forkhead and nuclear steroid receptor). We examined the extent to which all known genes containing each of these four transcription factor domains co-localize with UCRs (Figure 1). We found that a high proportion of these genes (163/1084; P-value 7.33e-11) are in genomic neighborhoods (<8 kb) of UCRs: more than 30% of all homeodomain-encoding genes have an UCR within 8 kbp (90/237; p-value 8.67e-11), and more than 55% have one within 100 kb (133/237, P-value 7.78e-11). The UCR association rates (the fraction of genes with an UCR closer than 8 kb, compared to the expected value) for genes encoding forkhead (8/31, P-value 6.6e-11), nuclear steroid receptor (9/38, P-value 2.81e-9) or zinc finger domains (56/751 P-value 8.12e-11) were noted as significant as well. These data provide strong evidence that the UCRs are spatially associated with genes encoding regulatory proteins.

**Table 1:** Over-representation of protein domains in genes flanking UCRs. Bonferroni-corrected and uncorrected Fisher Exact Test p-values are shown for the 16 most over-represented InterPro domains. Typical transcription factor domains (DNA binding domains) are indicated in bold. A full list of all InterPro domains with P-values is given in [Additional file 3].

***Domain description***	***INTERPRO ID***	***Fisher test P value***	***Corrected P value***
**HTH_lambrepressr**	IPR000047	6.40E-20	5.36E-17
**Homeobox**	IPR001356	1.60E-12	1.34E-09
**Antennapedia**	IPR001827	1.37E-10	1.15E-07
**Paired_box**	IPR001523	2.39E-05	2.00E-02
**HLH_basic**	IPR001092	2.40E-05	2.01E-02
**POU_domain**	IPR000327	3.06E-05	2.56E-02
**Homeo_OAR**	IPR003654	3.08E-05	2.58E-02
**TF_Fork_head**	IPR001766	6.15E-05	5.15E-02
**Znf_C4steroid**	IPR001628	7.45E-05	6.23E-02
**Hormone_rec_lig**	IPR000536	1.06E-04	8.86E-02
**HMG_12_box**	IPR000910	1.81E-04	1.51E-01
**Stdhrmn_receptor**	IPR001723	2.63E-04	2.20E-01
**COUP_TF**	IPR003068	7.62E-04	6.38E-01
**LIM**	IPR001781	1.10E-03	9.18E-01
**RtnoidX_receptor**	IPR000003	1.28E-03	1.07E+00
FN_III	IPR003961	2.57E-03	2.15E+00

### UCRs clusters encompass the entire gene loci of key developmental genes

**Figure 2:**
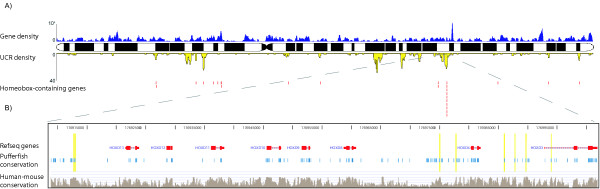
**Genomic distributions of UCRs and transcription factor genes**. **A**. Distribution of UCRs on human chromosome 2 is shown in yellow, and total gene density along the chromosome is shown in blue (top track). Note the lack of correlation between gene density and UCR density. Positions of homebox-domain containing genes locations are marked in red, and generally coincide with local maxima of UCR density. The remaining UCR density peaks coincide with genes for transcription factors belonging to structural classes other than homeobox. **B**. Close-up of a UCR cluster coinciding with the HoxD gene cluster. The HoxD cluster coincides with one of the larger UCR density peaks on chromosome 2, and is associated with nine UCRs. UCR locations are shaded in yellow.

**Figure 3:**
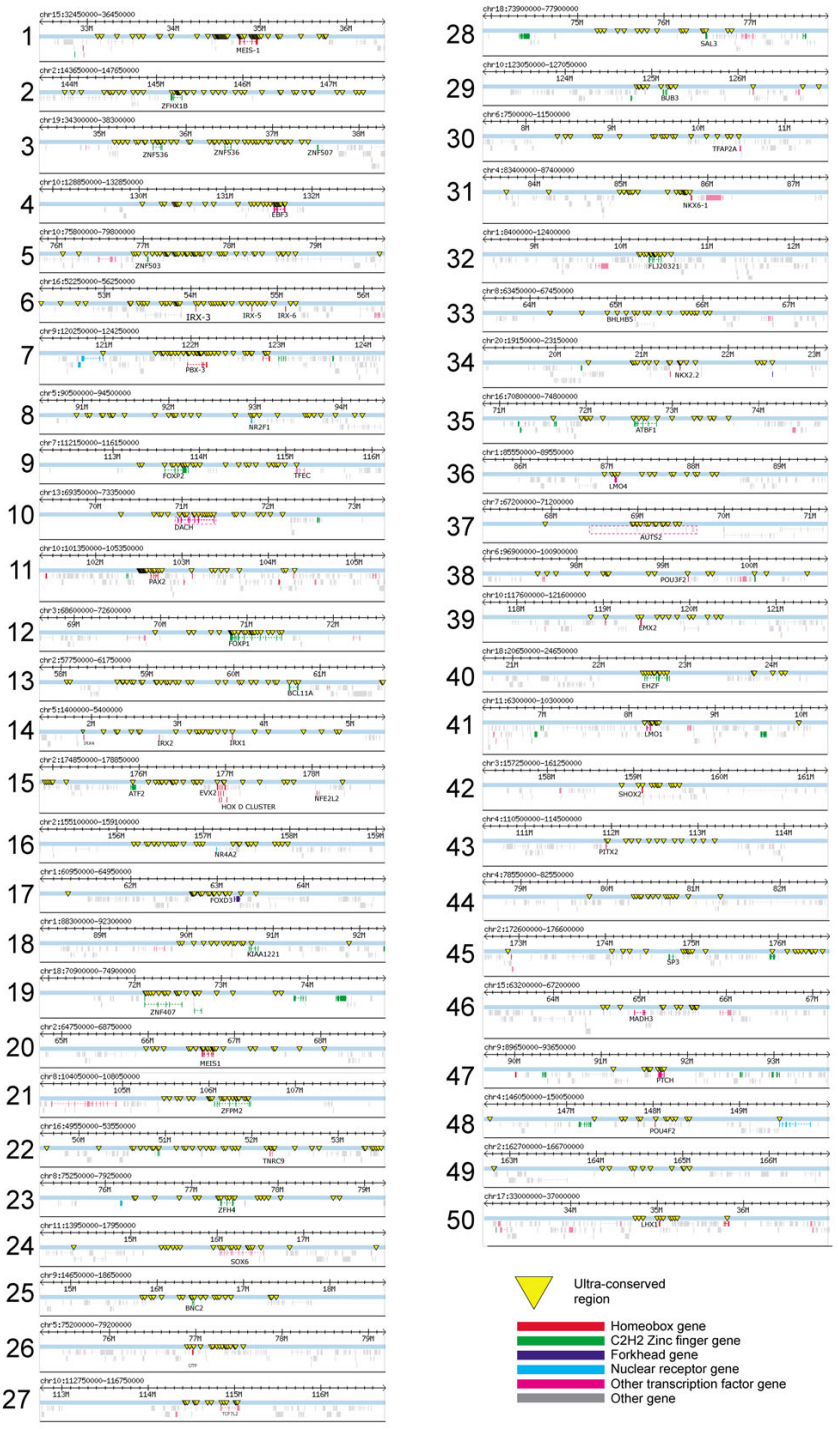
**Genomic landscape surrounding the most prominent UCR clusters in the human genome**. UCRs were counted by sliding a 500 kb window along the chromosomes. Overlapping UCR-containing windows were merged into a single cluster span. Each of the regions shows a 4 MB region around the corresponding UCR cluster. The cluster span coordinates correspond to the human genome NCBI build 33 (UCSC hg15, April 2003). Transcription factor genes are colored according to structural class. UCR clusters are visibly correlated with transcription factor genes; other developmental regulators that do not contain any of the probed protein domains were located manually (boxed), such as the autism susceptibility gene (chromosome 7, number 37) and the *DACH *gene (chromosome 13, number 10). The numbers correspond to annotations in [Additional file 6 and 7]. The figure was created with the help of the Bio::Graphics Perl library[27].

In order to visualize the distribution of UCR locations across the human genome, we generated a UCR density map for each chromosome [see Additional file 5]. Figure 2a shows such a map for chromosome 2. Visual inspection reveals an obvious qualitative tendency of UCRs to occur in large clusters, which was validated by a quantitative comparison of the distributions of nearest-neighbor distances between UCRs and a neutral background model (P-value 8.02e-16; Kolmogorov-Smirnov test). There is no observed correlation between regions of high gene density and UCRs, consistent with previously reported observations that larger conserved regions can be located in gene deserts [3]. As previously noted, many of the UCRs are adjacent to homeobox protein-encoding genes (Figure 1a, Figure 2b). It is interesting to note that the over-representation of UCRs near homeobox genes extends up to 300 kbp away from the transcription start site (Figure 1b). This is consistent with numerous observations that control regions need not be proximal to targeted genes, but can be located hundreds of kilobases from the transcription start site [3,7,16]. A similar trend is observed for UCRs near C2H2 zinc finger genes, with over-representation of UCRs extending up to 150 kbp away (Figure 1c). Large clusters of UCRs can span regions of several hundred kilobases around inferred target genes. For the 50 largest UCR clusters we generated comprehensive views of the chromosomal neighborhood (Figure 3). We find that 41 of the 50 clusters span one or more genes known to be expressed in embryonal development, including fundamental master regulator genes (*i.e*. the HoxD cluster, Nkx6.1, Nkx2.2 and Pbx3) [for detailed annotated lists of genes associated with UCR clusters, see Additional files 6 and 7]. To provide access to the entire set of UCRs, we have implemented a basic UCR browser  with links to the UCSC genome browser [17].

### Rare duplications of UCRs across evolution

**Figure 4:**
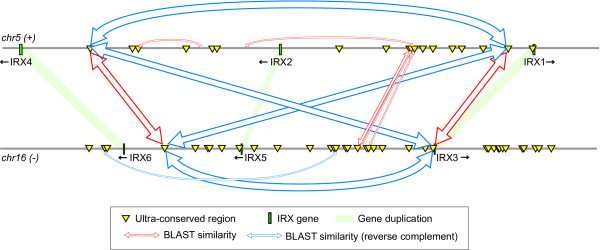
**Sets of UCRs sharing high sequence similarity are involved in regulation of related genes: the case of Iroquois gene clusters**. Four similarly positioned UCRs are located within the two Iroquois gene clusters at chromosomes 5 and 16. Block arrows indicate significant sequence similarity. The arrow width is inversely proportional to the alignment BLASTN E-value. There are additional shorter blocks of similarity between the two three-gene clusters; however, most UCRs have diverged between the two clusters, while still preserved across vertebrates.

We performed a global pairwise comparison of all UCRs, in order to determine if UCR duplication was common across evolution. We discovered only five sets of duplicated UCRs, all of which are adjacent to corresponding duplicated genes. For example, duplicated UCRs are present in the introns of SOX5 (on chromosome 12) and SOX6 (on chromosome 11), two highly similar genes involved in chondrocyte differentiation [18]. Of special interest is the conservation of UCRs in the Iroquois (*IRX*) gene clusters. *IRX *genes are situated in two clusters of three genes each, present on human chromosomes 5 and 16 [19]. Similarly positioned arrays of UCRs are present in each of the four intergenic regions between the IRX genes (Figure 4). The great majority of UCRs, while conserved across vertebrate evolution, show no similarity between the clusters within the species. An intriguing exception is the set of four UCRs that are highly similar in both cluster position and nucleotide sequence.

## Discussion

The human genome contains numerous ultra-conserved regulatory sequences that are shared broadly across vertebrates. *These UCRs occur in arrays of highly conserved regulatory elements spanning large chromosomal regions*. The clusters are co-localized with genes encoding key proteins for the regulation of development, with a particular correlation with genes encoding transcription factors. The strength of association between UCRs and diverse classes of DNA binding transcription factors validates that a relatively simple definition of UCRs captures a biologically meaningful set of functional sequences. The presence of non-coding UCRs is predictive for the presence of genes implicated in development, differentiation and malignancies. The list presented in [Additional file 6] hints at potentially crucial roles of currently uncharacterized transcription factor genes, while the collection of reported UCRs provides a wealth of regulatory locations for further study.

Exceptional mechanisms are brought to bear to retain UCRs over hundreds of millions of years of parallel evolution. UCRs are more strongly conserved than sequences encoding identical proteins, and exhibit sequence identity exceeding essentially all known cis-regulatory sequences. The retention properties suggest that UCRs have important functions in the vertebrate genome.

The observed UCRs could fall into multiple functional categories, including enhancers of transcription, regulators of chromatin structure and unknown genes for non-coding transcripts. A small subset of UCRs have been identified previously as enhancers of transcription [7,3].

The high conservation and length of UCRs compared to binding sites for single transcription factors suggests that the mode of regulation must involve more than the binding of small number of transcription factors. Homeotypic clusters of binding sites, as seen in developmental genes in *Drosophila melanogaster *[20], represent one regulatory mechanism that could explain the occurrence of long, conserved non-coding regions. However, as transcription factors tolerate considerable variation between functional binding sites, a homeotypic cluster of binding sites as such cannot warrant the extreme level of conservation observed in UCRs. Alternatively, the recent emergence of the role of microRNAs in regulation suggests that there could be additional non-coding genes in the human genome, perhaps at the sites of ultra-conservation.

The clustering of UCRs suggests that UCR-mediated transcriptional regulation may involve molecular events on a greater scale, possibly involving chromatin structure. This potential link to chromatin structure is suggested by the striking pattern of UCRs in the *IRX *gene clusters. Most of the UCRs have no similarity between the two clusters, with the exception of a set of four UCRs that have retained both mutual sequence similarity and spatial position (Figure 4). It is tempting to assume that the retention of their mutual similarity is a consequence of *IRX *cluster co-regulation, the mechanism of which remains unknown.

Based on the preservation of nearly identical sequences over ~450 million years of vertebrate evolution, it is reasonable to postulate the influence of exceptional biochemical mechanisms. Numerous hypotheses could account for the observed data, broadly falling into two categories – active mechanism(s) resulting in the decrease of mutational frequency in UCRs, or negative pressure consistent with evolutionary selection against such mutations. Given the breadth of possibilities, we leave postulation until further data emerges.

## Conclusion

Since Bejerano *et al*.[9] focused on larger regions (200 bp) of perfect nucleotide identity compared to our more permissive settings (95% sequence identity over 50 bp), the genomic arrangement of UCR-containing regions with respect to their presumptive target genes was not fully realized. Our findings include critical new information about UCR clusters, particularly with regards to patterns of conservation, their genomic organization, and the insights they provide into potential chromatin regulating mechanisms. These mysterious regions retained over hundreds of millions of years of evolution appear to contribute to a novel mechanism of developmental regulation. Detailed studies of UCRs that will ensue from the discoveries reported here promise to advance our understanding of vertebrate development.

## Methods

### Definition of UCRs applied in this study

We defined UCRs as non-protein coding genomic regions having a sequence identity > 95% over a 50 bp sliding window of length in human/mouse comparison (based on the tight alignments track from the UCSC genome browser database[17], using human and mouse assemblies hg15 and mm3, respectively). As a further constraint, an UCR must overlap with sequences conserved between the human and pufferfish genomes, as defined in the UCSC genome browser databases (a BLAT [21] alignment between human and pufferfish with a minimum BLAT score of 20). In order to avoid inclusion of coding sequence, we required that a UCR must not overlap a mouse or human cDNA mapped to the genome (based on cDNA tracks from from the UCSC genome browser database[17]) or overlap putative coding regions predicted by GenScan [22].

### Calculation of UCR and gene distributions

The distribution of UCRs in the genome was calculated by counting the number of UCRs within a 500 kilobase (kb) window which was progressively slid over each chromosome in 100 kb intervals. The same approach was used to estimate the gene density; specifically by summing the number of bases within the window that aligned with human mRNA (from the UCSC Genome Browser database).

### Gene-UCR distance calculation

Distances between a given gene and UCR on the same chromosome were defined as the shortest distance between the starting points and/or endpoints of UCR and gene in the human genome (UCSC assembly hg15), using EnsEMBL [23] gene annotation. Genes based solely on ESTs or computational predictions were not included.

### Estimation of significance of Gene-UCR distances

The distances from genes within a set (for instance, all forkhead domain-containing genes) to the closest UCRs were calculated as above. The expected fraction of gene-UCR distances smaller than 8 kb was estimated by simulation: UCR genome coordinates were randomly chosen and distances measured as above. The simulation process was repeated 1000 times and the average fraction reported. In order to estimate if the observed distribution was significantly different from the expected, we used the chi-squared test.

### Estimation of domain over-representation in genes closest to UCRs

For each UCR, the closest upstream and downstream gene within 2 Mbp was identified (UCRs inside introns of genes were analyzed separately). EnsEMBL InterPro [14] domain annotation was used to tabulate a contingency table consisting of the positive sample counts (number of genes in the set containing a certain domain), negative sample counts (number of remaining genes in the set), background positives (number of genes containing the same domain in the genome) and background negatives (remaining genes). For clarity, a given gene was only counted once, and multiple occurrences of the same domain within the same protein were not counted.

For each domain found in the UCR-proximal genes, we tested the null-hypothesis that the sample and background sets are drawn from the same population versus the alternative hypothesis that the sample set has a higher frequency of the domain, using Fisher's Exact Test [24] from the R statistical package . Since the number of tests is considerable, we corrected for multiple sampling using the conservative Bonferroni method [15], in which the number of tests is multiplied with the P-value from the Fisher test with the number of unique domains tested (837). An analogous analysis was performed with genes containing one or more UCRs within their introns [see Additional file 4].

### Estimation of clustering tendency

We used the distances between consecutive UCRs as a statistic indicating clustering. A neutral background distance distribution was created by assigning UCRs genome coordinates randomly, and subsequently measuring distances between consecutive UCRs. This process was repeated 1000 times. We compared the distance distribution between naturally occurring UCRs and the background using the Kolmogorov-Smirnov test [25], which assigns a probability that two distributions are similarly shaped.

### UCR sequence similarity analysis

All possible pairs of UCRs were aligned using NCBI BLASTN [26] with standard settings. For any pair to be reported as near-identical, we required an HSP of at least 50 bp and a pairwise sequence identity exceeding 75%.

## Abbreviations

UCR – ultraconserved non-coding region; bp – basepairs; kbp – 10^3 ^base pairs

## Authors' contributions

AS collected the data and performed most steps of bioinformatic and statistical analysis presented in the paper. He produced all the Figures in the paper and Table 1, and co-wrote the manuscript. PB made initial analyses of putative regulatory elements on selected genes involved in neural tube development. He discovered a number of super-conserved regions in the process, which helped create the rules for their genome-wide computational detection. He also co-wrote the first versions of the manuscript. SB participated in the annotation of the gene set and in the creation of software for the visualization of results. PE prepared genome sequence and annotation data for human, mouse and pufferfish. He and AS designed the statistical tests applied in the study. JK participated in the initial analyses and data extraction with PB. WWW participated in result interpretation, design of statistical tests, and writing later versions of the manuscript. JE initiated and co-supervised the study, which has the roots in his research in developmental neurobiology. He also co-wrote the manuscript. BL designed and supervised the bioinformatic study, developed the initial framework for the analysis of the genomic sequences, made an independent observation about high incidence of clustering of super-conserved regions around genes encoding DNA-binding proteins, and annotated the UCR clusters with co-localizing genes. He also co-wrote the manuscript.

## Supplementary Material

Additional File 1 **Genescape around 50 randomly selected UCRs**. Selected UCRs are shown as yellow triangles, other UCRs as light yellow triangles. Genes are colored after domain (red = Homeobox, green = C2H2 Zink fingers in green, pink = Nuclear receptors, Blue = forkhead). Click here for file

Additional File 2 **Genescape around 50 randomly selected genes**. UCRs are shown as as light yellow triangles. Color coding of genes as above. Click here for file

Additional File 3 **Complete list of protein domains in genes flanking UCRs**. Each tested domain is listed along with corrected and uncorrected P-value as in Table 1. Click here for file

Additional File 4 **Complete list of protein domains in genes with UCR(s) in intron(s) **Each tested domain is listed along with corrected and uncorrected P-value as in Table 1. Click here for file

Additional File 5 **UCR distribution in the human genome **UCR density (pink) and gene density (blue) is shown for each chromosome. Densities are calculated as described in Methods. Click here for file

Additional File 6 **Genes associated with enumerated UCR clusters from Figure **3. UCRs were counted by sliding a 500 kb window along the chromosomes. Overlapping UCR-containing windows were merged into a single cluster span. The cluster span coordinates correspond to the human genome NCBI build 33 (UCSC hg15, April 2003). A more exhaustive list is found in [Additional file 7] Click here for file

Additional File 7 **Extended list of UCR clusters **An extended, but less annotated, version of in [Additional file 6] Click here for file
