# Supplementary figures and images for: Arrays of ultraconserved non-coding regions span the loci of key developmental genes in vertebrate genomes
Source: BMC Genomics. 2004 Dec 21;5:99. doi: 10.1186/1471-2164-5-99 (PMC544600; doi:10.1186/1471-2164-5-99)

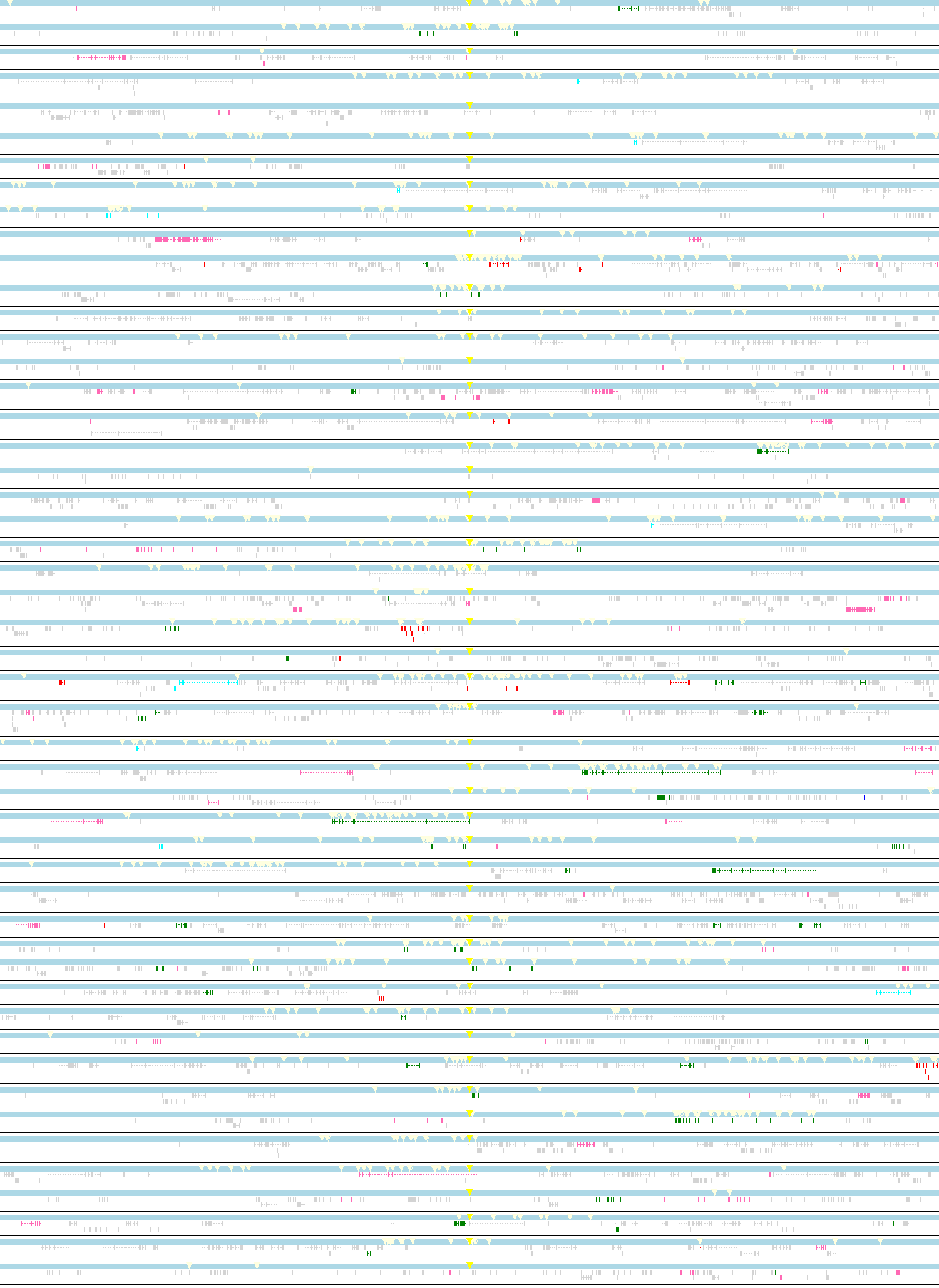

Supplement: Additional File 1 — Genescape around 50 randomly selected UCRs. Selected UCRs are shown as yellow triangles, other UCRs as light yellow triangles. Genes are colored after domain (red = Homeobox, green = C2H2 Zink fingers in green, pink = Nuclear receptors, Blue = forkhead). [file 1471-2164-5-99-S1.png]

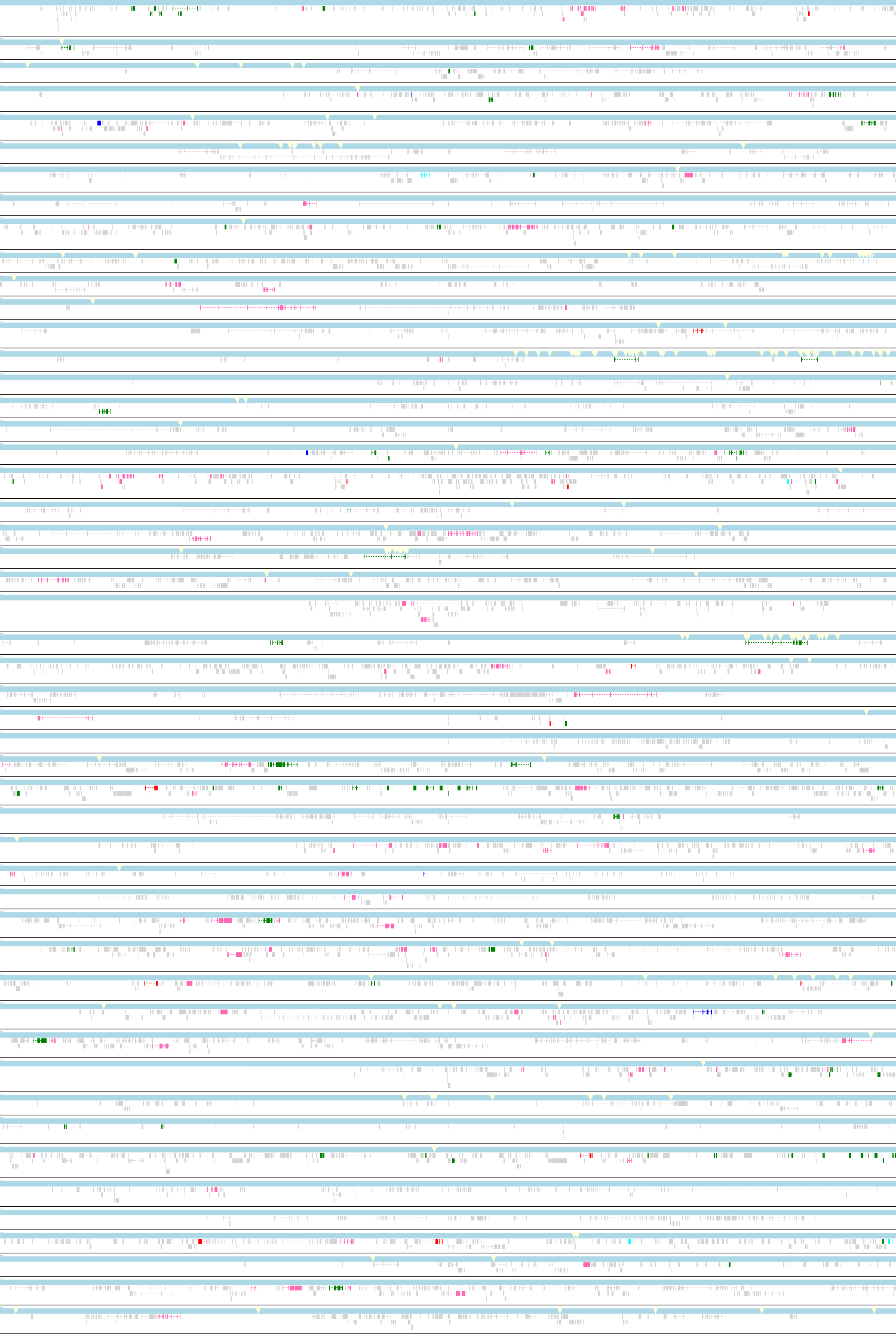

Supplement: Additional File 2 — Genescape around 50 randomly selected genes. UCRs are shown as as light yellow triangles. Color coding of genes as above. [file 1471-2164-5-99-S2.png]

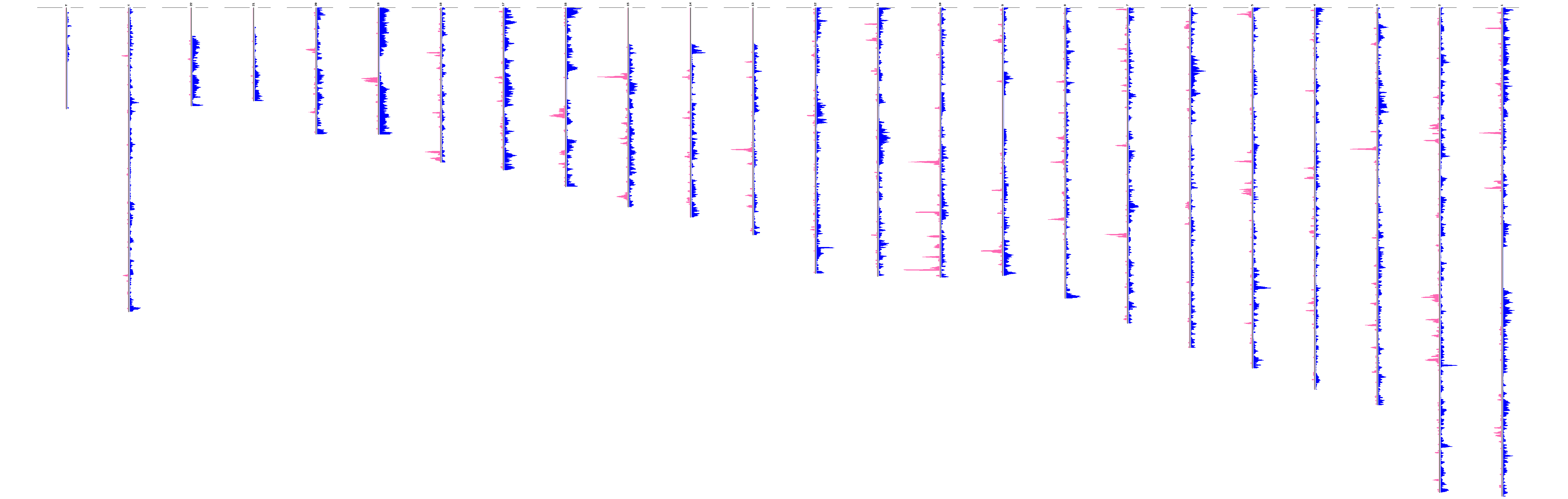

Supplement: Additional File 5 — UCR distribution in the human genome UCR density (pink) and gene density (blue) is shown for each chromosome. Densities are calculated as described in Methods. [file 1471-2164-5-99-S5.png]
