# Supplementary material for: Arrays of ultraconserved non-coding regions span the loci of key developmental genes in vertebrate genomes
Source: BMC Genomics. 2004 Dec 21;5:99. doi: 10.1186/1471-2164-5-99 (PMC544600; doi:10.1186/1471-2164-5-99)
Supplement: Additional File 3 — Complete list of protein domains in genes flanking UCRs. Each tested domain is listed along with corrected and uncorrected P-value as in Table 1. [file 1471-2164-5-99-S3.html]

Table S2


Complete list of protein domains in genes flanking �UCRs.

| ``` domain label ``` | ``` interpro_id ``` | ``` fisher test p value ``` | ``` Bonferroni-corrected p value ``` |
| --- | --- | --- | --- |
| ``` HTH_lambrepressr ``` | ``` IPR000047 ``` | ``` -3.76283448844106e-11 ``` | ``` -3.14949246682517e-08 ``` |
| ``` Homeobox ``` | ``` IPR001356 ``` | ``` 1.5967227540159e-12 ``` | ``` 1.33645694511131e-09 ``` |
| ``` Antennapedia ``` | ``` IPR001827 ``` | ``` 1.37164613001062e-10 ``` | ``` 1.14806781081889e-07 ``` |
| ``` Paired_box ``` | ``` IPR001523 ``` | ``` 2.38542461701297e-05 ``` | ``` 0.0199660040443986 ``` |
| ``` HLH_basic ``` | ``` IPR001092 ``` | ``` 2.40019747963505e-05 ``` | ``` 0.0200896529045454 ``` |
| ``` POU_domain ``` | ``` IPR000327 ``` | ``` 3.06015942782567e-05 ``` | ``` 0.0256135344109009 ``` |
| ``` Homeo_OAR ``` | ``` IPR003654 ``` | ``` 3.08228067508187e-05 ``` | ``` 0.0257986892504353 ``` |
| ``` TF_Fork_head ``` | ``` IPR001766 ``` | ``` 6.14712746318524e-05 ``` | ``` 0.0514514568668605 ``` |
| ``` Znf_C4steroid ``` | ``` IPR001628 ``` | ``` 7.44900903884016e-05 ``` | ``` 0.0623482056550921 ``` |
| ``` Hormone_rec_lig ``` | ``` IPR000536 ``` | ``` 0.000105810728518341 ``` | ``` 0.0885635797698514 ``` |
| ``` HMG_12_box ``` | ``` IPR000910 ``` | ``` 0.000180568985135632 ``` | ``` 0.151136240558524 ``` |
| ``` Stdhrmn_receptor ``` | ``` IPR001723 ``` | ``` 0.000263403866121115 ``` | ``` 0.220469035943373 ``` |
| ``` COUP_TF ``` | ``` IPR003068 ``` | ``` 0.000762193033902125 ``` | ``` 0.637955569376079 ``` |
| ``` LIM ``` | ``` IPR001781 ``` | ``` 0.00109695500811902 ``` | ``` 0.91815134179562 ``` |
| ``` RtnoidX_receptor ``` | ``` IPR000003 ``` | ``` 0.00128148208289836 ``` | ``` 1.07260050338593 ``` |
| ``` FN_III ``` | ``` IPR003961 ``` | ``` 0.00256864551927216 ``` | ``` 2.1499562996308 ``` |
| ``` Antifreeze_1 ``` | ``` IPR000104 ``` | ``` 0.0027402854722568 ``` | ``` 2.29361894027894 ``` |
| ``` DM_DNA-binding ``` | ``` IPR001275 ``` | ``` 0.00338815738652198 ``` | ``` 2.8358877325189 ``` |
| ``` Engrailed ``` | ``` IPR000747 ``` | ``` 0.00410299480604959 ``` | ``` 3.43420665266351 ``` |
| ``` KV14channel ``` | ``` IPR004051 ``` | ``` 0.00685088487288377 ``` | ``` 5.73419063860372 ``` |
| ``` SNF2_N ``` | ``` IPR000330 ``` | ``` 0.00796103391557412 ``` | ``` 6.66338538733554 ``` |
| ``` PRO_rich ``` | ``` IPR000694 ``` | ``` 0.0095723608763778 ``` | ``` 8.01206605352822 ``` |
| ``` TF_AP2 ``` | ``` IPR004979 ``` | ``` 0.0104602991292443 ``` | ``` 8.75527037117748 ``` |
| ``` NLS_BP ``` | ``` IPR001472 ``` | ``` 0.0126210045463442 ``` | ``` 10.5637808052901 ``` |
| ``` MH1 ``` | ``` IPR004863 ``` | ``` 0.0145821839721481 ``` | ``` 12.205287984688 ``` |
| ``` FnIII_subd ``` | ``` IPR003962 ``` | ``` 0.0155578135102915 ``` | ``` 13.021889908114 ``` |
| ``` TGFb_N ``` | ``` IPR001111 ``` | ``` 0.0199357637679727 ``` | ``` 16.6862342737932 ``` |
| ``` Otx_TF ``` | ``` IPR003025 ``` | ``` 0.0211058871573008 ``` | ``` 17.6656275506608 ``` |
| ``` Ribosomal_L30 ``` | ``` IPR000517 ``` | ``` 0.0319049154699698 ``` | ``` 26.7044142483647 ``` |
| ``` TF_COE ``` | ``` IPR003523 ``` | ``` 0.0337428351621034 ``` | ``` 28.2427530306805 ``` |
| ``` ZU5 ``` | ``` IPR000906 ``` | ``` 0.0341350726639990 ``` | ``` 28.5710558197672 ``` |
| ``` DEAH_box ``` | ``` IPR002464 ``` | ``` 0.035261824946743 ``` | ``` 29.5141474804239 ``` |
| ``` TF_T-box ``` | ``` IPR001699 ``` | ``` 0.0375379669192033 ``` | ``` 31.4192783113732 ``` |
| ``` Znf_PHD ``` | ``` IPR001965 ``` | ``` 0.0394220065672841 ``` | ``` 32.9962194968168 ``` |
| ``` Methyl-CpG_bind ``` | ``` IPR001739 ``` | ``` 0.0423819872850746 ``` | ``` 35.4737233576074 ``` |
| ``` StAR ``` | ``` IPR000799 ``` | ``` 0.0485627553598298 ``` | ``` 40.6470262361775 ``` |
| ``` Eggshell ``` | ``` IPR002952 ``` | ``` 0.0504025180391655 ``` | ``` 42.1869075987815 ``` |
| ``` Bmbsn_receptor ``` | ``` IPR001556 ``` | ``` 0.051526169567901 ``` | ``` 43.1274039283331 ``` |
| ``` Transposase_22 ``` | ``` IPR004244 ``` | ``` 0.0543029416748028 ``` | ``` 45.4515621818099 ``` |
| ``` Znf_C2H2 ``` | ``` IPR007087 ``` | ``` 0.0562189756091551 ``` | ``` 47.0552825848628 ``` |
| ``` ThyrH_receptor ``` | ``` IPR001728 ``` | ``` 0.0615352288872268 ``` | ``` 51.5049865786088 ``` |
| ``` TLE_N ``` | ``` IPR005617 ``` | ``` 0.0652475035708477 ``` | ``` 54.6121604887995 ``` |
| ``` Ribosomal_7A ``` | ``` IPR001921 ``` | ``` 0.0653798275302866 ``` | ``` 54.7229156428499 ``` |
| ``` CytC_heme_bind ``` | ``` IPR000345 ``` | ``` 0.0653913946024809 ``` | ``` 54.7325972822765 ``` |
| ``` Rtnoid_receptor ``` | ``` IPR003078 ``` | ``` 0.0723705468996525 ``` | ``` 60.5741477550091 ``` |
| ``` M+channel_nlg ``` | ``` IPR005820 ``` | ``` 0.0742714794464043 ``` | ``` 62.1652282966404 ``` |
| ``` Ser_thr_pkinase ``` | ``` IPR002290 ``` | ``` 0.0802914709231863 ``` | ``` 67.2039611627069 ``` |
| ``` Ribosomal_S5 ``` | ``` IPR000851 ``` | ``` 0.0824056550965477 ``` | ``` 68.9735333158104 ``` |
| ``` RFX_DNA_binding ``` | ``` IPR003150 ``` | ``` 0.0835099050272481 ``` | ``` 69.8977905078067 ``` |
| ``` ARID ``` | ``` IPR001606 ``` | ``` 0.083988590394952 ``` | ``` 70.2984501605748 ``` |
| ``` Sec7 ``` | ``` IPR000904 ``` | ``` 0.0963420596946293 ``` | ``` 80.6383039644047 ``` |
| ``` Glucagon ``` | ``` IPR000532 ``` | ``` 0.103091143919464 ``` | ``` 86.2872874605914 ``` |
| ``` TGFb ``` | ``` IPR001839 ``` | ``` 0.107121894843718 ``` | ``` 89.661025984192 ``` |
| ``` CPase_L_D2 ``` | ``` IPR005479 ``` | ``` 0.109380890076874 ``` | ``` 91.5518049943435 ``` |
| ``` SET ``` | ``` IPR001214 ``` | ``` 0.115279011743251 ``` | ``` 96.4885328291011 ``` |
| ``` Zeta_haem ``` | ``` IPR002340 ``` | ``` 0.119912891466825 ``` | ``` 100.367090157733 ``` |
| ``` Chromo ``` | ``` IPR000953 ``` | ``` 0.122149146438040 ``` | ``` 102.238835568639 ``` |
| ``` Sulfatase ``` | ``` IPR000917 ``` | ``` 0.123053120891109 ``` | ``` 102.995462185858 ``` |
| ``` TF_Maf ``` | ``` IPR004826 ``` | ``` 0.123758356863884 ``` | ``` 103.585744695071 ``` |
| ``` PAS_domain ``` | ``` IPR000014 ``` | ``` 0.144565145889832 ``` | ``` 121.001027109789 ``` |
| ``` Nucorph_receptor ``` | ``` IPR003070 ``` | ``` 0.145302415521760 ``` | ``` 121.618121791713 ``` |
| ``` Ribosomal_L7Ae ``` | ``` IPR004037 ``` | ``` 0.152084861877783 ``` | ``` 127.295029391704 ``` |
| ``` Bromodomain ``` | ``` IPR001487 ``` | ``` 0.153394653542893 ``` | ``` 128.391325015401 ``` |
| ``` EFTU_D2 ``` | ``` IPR004161 ``` | ``` 0.156332528132560 ``` | ``` 130.850326046953 ``` |
| ``` NPY_receptor ``` | ``` IPR000611 ``` | ``` 0.163513753160076 ``` | ``` 136.861011394984 ``` |
| ``` EFTU_Cterm ``` | ``` IPR004160 ``` | ``` 0.167337213436837 ``` | ``` 140.061247646633 ``` |
| ``` Znf_CXXC ``` | ``` IPR002857 ``` | ``` 0.167535884154707 ``` | ``` 140.22753503749 ``` |
| ``` Death ``` | ``` IPR000488 ``` | ``` 0.168441142848542 ``` | ``` 140.98523656423 ``` |
| ``` eRF1_3 ``` | ``` IPR005142 ``` | ``` 0.174367409126274 ``` | ``` 145.945521438691 ``` |
| ``` TF_bZIP ``` | ``` IPR004827 ``` | ``` 0.174531984124426 ``` | ``` 146.083270712145 ``` |
| ``` IPT_TIG ``` | ``` IPR002909 ``` | ``` 0.180866497502800 ``` | ``` 151.385258409844 ``` |
| ``` Int_Cl_channel ``` | ``` IPR002946 ``` | ``` 0.190291138102309 ``` | ``` 159.273682591633 ``` |
| ``` EGF_II ``` | ``` IPR001438 ``` | ``` 0.193583727146726 ``` | ``` 162.02957962181 ``` |
| ``` Helicase_C ``` | ``` IPR001650 ``` | ``` 0.194343762612360 ``` | ``` 162.665729306545 ``` |
| ``` TYR_phosphatase ``` | ``` IPR000387 ``` | ``` 0.209343379664732 ``` | ``` 175.220408779381 ``` |
| ``` Znf_GATA ``` | ``` IPR000679 ``` | ``` 0.213418632882726 ``` | ``` 178.631395722842 ``` |
| ``` Rhodanese-like ``` | ``` IPR001763 ``` | ``` 0.215407627519614 ``` | ``` 180.296184233917 ``` |
| ``` CSA_PPIase ``` | ``` IPR002130 ``` | ``` 0.217691740478216 ``` | ``` 182.207986780267 ``` |
| ``` cAMP_dep_PKI ``` | ``` IPR004171 ``` | ``` 0.225454583164649 ``` | ``` 188.705486108811 ``` |
| ``` LRR_Nterm ``` | ``` IPR000372 ``` | ``` 0.229141036528791 ``` | ``` 191.791047574598 ``` |
| ``` Ribosomal_S5_C ``` | ``` IPR005324 ``` | ``` 0.232032874029031 ``` | ``` 194.211515562299 ``` |
| ``` F-box ``` | ``` IPR001810 ``` | ``` 0.233226120566795 ``` | ``` 195.210262914407 ``` |
| ``` Mit_carrier ``` | ``` IPR002067 ``` | ``` 0.233235673832655 ``` | ``` 195.218258997932 ``` |
| ``` Olfac_like ``` | ``` IPR003112 ``` | ``` 0.236785310414865 ``` | ``` 198.189304817242 ``` |
| ``` IQ_region ``` | ``` IPR000048 ``` | ``` 0.242702472558316 ``` | ``` 203.141969531311 ``` |
| ``` N6_Mtase ``` | ``` IPR002052 ``` | ``` 0.248878185967593 ``` | ``` 208.311041654875 ``` |
| ``` PX ``` | ``` IPR001683 ``` | ``` 0.252532138281908 ``` | ``` 211.369399741957 ``` |
| ``` UBX ``` | ``` IPR001012 ``` | ``` 0.260273133740274 ``` | ``` 217.848612940609 ``` |
| ``` Tyr_PP ``` | ``` IPR000242 ``` | ``` 0.264443304155547 ``` | ``` 221.339045578193 ``` |
| ``` Keratin_I ``` | ``` IPR002957 ``` | ``` 0.264618314890418 ``` | ``` 221.48552956328 ``` |
| ``` Ion_trans ``` | ``` IPR005821 ``` | ``` 0.268707437732990 ``` | ``` 224.908125382513 ``` |
| ``` eIF5_eIF2B ``` | ``` IPR002735 ``` | ``` 0.273382520151506 ``` | ``` 228.821169366811 ``` |
| ``` RasGAP ``` | ``` IPR001936 ``` | ``` 0.283777739532565 ``` | ``` 237.521967988757 ``` |
| ``` AAA_ATPase_centr ``` | ``` IPR003959 ``` | ``` 0.288620650592472 ``` | ``` 241.575484545899 ``` |
| ``` Involucrin_rpt ``` | ``` IPR000354 ``` | ``` 0.307207199521241 ``` | ``` 257.132425999279 ``` |
| ``` EGF_like ``` | ``` IPR006209 ``` | ``` 0.312200466963487 ``` | ``` 261.311790848439 ``` |
| ``` ANF_receptor ``` | ``` IPR001828 ``` | ``` 0.317069187948523 ``` | ``` 265.386910312914 ``` |
| ``` EF_GTPbind ``` | ``` IPR000795 ``` | ``` 0.317575875616813 ``` | ``` 265.811007891272 ``` |
| ``` Znf_C5HC2 ``` | ``` IPR004198 ``` | ``` 0.318346473121693 ``` | ``` 266.455998002857 ``` |
| ``` Kringle ``` | ``` IPR000001 ``` | ``` 0.330480882989504 ``` | ``` 276.612499062215 ``` |
| ``` DS_phosphatase ``` | ``` IPR000340 ``` | ``` 0.334876948567354 ``` | ``` 280.292005950875 ``` |
| ``` Znf_MYND ``` | ``` IPR002893 ``` | ``` 0.353528412112436 ``` | ``` 295.903280938109 ``` |
| ``` Znf_Nrecognin ``` | ``` IPR003126 ``` | ``` 0.360529634723196 ``` | ``` 301.763304263315 ``` |
| ``` DAG_PE-bind ``` | ``` IPR002219 ``` | ``` 0.362928097916760 ``` | ``` 303.770817956328 ``` |
| ``` Znf_DHHC ``` | ``` IPR001594 ``` | ``` 0.376288703185935 ``` | ``` 314.953644566628 ``` |
| ``` Mitoch_carrier ``` | ``` IPR001993 ``` | ``` 0.383563147483742 ``` | ``` 321.042354443892 ``` |
| ``` RA_domain ``` | ``` IPR000159 ``` | ``` 0.386516125309378 ``` | ``` 323.513996883949 ``` |
| ``` Ribosomal_S2 ``` | ``` IPR001865 ``` | ``` 0.398709087349451 ``` | ``` 333.71950611149 ``` |
| ``` Znf_AN1 ``` | ``` IPR000058 ``` | ``` 0.400103881808465 ``` | ``` 334.886949073685 ``` |
| ``` Zn_carbOpept ``` | ``` IPR000834 ``` | ``` 0.403539572204393 ``` | ``` 337.762621935077 ``` |
| ``` K_tetra ``` | ``` IPR003131 ``` | ``` 0.417018327613684 ``` | ``` 349.044340212654 ``` |
| ``` Helicase_dom ``` | ``` IPR007502 ``` | ``` 0.420744503901252 ``` | ``` 352.163149765348 ``` |
| ``` cNMP_binding ``` | ``` IPR000595 ``` | ``` 0.437141991162852 ``` | ``` 365.887846603307 ``` |
| ``` VPS9 ``` | ``` IPR003123 ``` | ``` 0.437230474050624 ``` | ``` 365.961906780372 ``` |
| ``` T_SNARE ``` | ``` IPR000727 ``` | ``` 0.442356761821634 ``` | ``` 370.252609644708 ``` |
| ``` kazal ``` | ``` IPR002350 ``` | ``` 0.453674935794328 ``` | ``` 379.725921259853 ``` |
| ``` Calponin-like ``` | ``` IPR001715 ``` | ``` 0.46211457933177 ``` | ``` 386.789902900692 ``` |
| ``` GCN5acetyltransf ``` | ``` IPR000182 ``` | ``` 0.463513862896811 ``` | ``` 387.961103244631 ``` |
| ``` Cadherin ``` | ``` IPR002126 ``` | ``` 0.464087813853181 ``` | ``` 388.441500195112 ``` |
| ``` BTB_POZ ``` | ``` IPR000210 ``` | ``` 0.467014161460973 ``` | ``` 390.890853142834 ``` |
| ``` ARF/SAR ``` | ``` IPR006689 ``` | ``` 0.470001673160987 ``` | ``` 393.391400435746 ``` |
| ``` eIF5C ``` | ``` IPR003307 ``` | ``` 0.472060709297995 ``` | ``` 395.114813682422 ``` |
| ``` EGF_Ca ``` | ``` IPR001881 ``` | ``` 0.475517252815441 ``` | ``` 398.007940606524 ``` |
| ``` FHA ``` | ``` IPR000253 ``` | ``` 0.484189382555794 ``` | ``` 405.2665131992 ``` |
| ``` Ras_trnsfrmng ``` | ``` IPR001806 ``` | ``` 0.490759379800442 ``` | ``` 410.76560089297 ``` |
| ``` LRR_Cterm ``` | ``` IPR000483 ``` | ``` 0.496815561823549 ``` | ``` 415.834625246311 ``` |
| ``` RhoGAP ``` | ``` IPR000198 ``` | ``` 0.498256716695625 ``` | ``` 417.040871874238 ``` |
| ``` ANK ``` | ``` IPR002110 ``` | ``` 0.500892755602952 ``` | ``` 419.247236439671 ``` |
| ``` TF_JmjC ``` | ``` IPR003347 ``` | ``` 0.504361903210502 ``` | ``` 422.15091298719 ``` |
| ``` Synaptobrevin ``` | ``` IPR001388 ``` | ``` 0.504736538644673 ``` | ``` 422.464482845591 ``` |
| ``` Tropomyosin ``` | ``` IPR000533 ``` | ``` 0.51757223786549 ``` | ``` 433.207963093415 ``` |
| ``` Prot_kinase ``` | ``` IPR000719 ``` | ``` 0.528830258942 ``` | ``` 442.630926734454 ``` |
| ``` UBQ_conjugat ``` | ``` IPR000608 ``` | ``` 0.53291049630765 ``` | ``` 446.046085409503 ``` |
| ``` ABC_transporter ``` | ``` IPR003439 ``` | ``` 0.5334334826443 ``` | ``` 446.483824973279 ``` |
| ``` Rib_prot_L13 ``` | ``` IPR005822 ``` | ``` 0.535391143630648 ``` | ``` 448.122387218852 ``` |
| ``` M_repeat ``` | ``` IPR003345 ``` | ``` 0.540175371555227 ``` | ``` 452.126785991725 ``` |
| ``` Asx_hydroxyl ``` | ``` IPR000152 ``` | ``` 0.54448371805606 ``` | ``` 455.732872012922 ``` |
| ``` UCH-2 ``` | ``` IPR001394 ``` | ``` 0.552269967590178 ``` | ``` 462.249962872979 ``` |
| ``` Aspprotease_site ``` | ``` IPR001969 ``` | ``` 0.561711841159398 ``` | ``` 470.152811050416 ``` |
| ``` YKase_receptorV ``` | ``` IPR001426 ``` | ``` 0.564149477409996 ``` | ``` 472.193112592167 ``` |
| ``` Hydrolase ``` | ``` IPR005834 ``` | ``` 0.577209799755047 ``` | ``` 483.124602394974 ``` |
| ``` Sema ``` | ``` IPR001627 ``` | ``` 0.579741129283518 ``` | ``` 485.243325210305 ``` |
| ``` Znf_UBP ``` | ``` IPR001607 ``` | ``` 0.591128773155126 ``` | ``` 494.77478313084 ``` |
| ``` Tyr_pkinase ``` | ``` IPR001245 ``` | ``` 0.605440841694123 ``` | ``` 506.753984497981 ``` |
| ``` Ig_MHC ``` | ``` IPR003006 ``` | ``` 0.610336198229383 ``` | ``` 510.851397917994 ``` |
| ``` SAM ``` | ``` IPR001660 ``` | ``` 0.610723469848524 ``` | ``` 511.175544263215 ``` |
| ``` Regl_Gprotein ``` | ``` IPR000342 ``` | ``` 0.614144094435922 ``` | ``` 514.038607042867 ``` |
| ``` Tub_tyr_lygase ``` | ``` IPR004344 ``` | ``` 0.616439020490999 ``` | ``` 515.959460150966 ``` |
| ``` Laminin_G ``` | ``` IPR001791 ``` | ``` 0.618771109265995 ``` | ``` 517.911418455638 ``` |
| ``` DUF741 ``` | ``` IPR007994 ``` | ``` 0.62647518465289 ``` | ``` 524.359729554469 ``` |
| ``` Aden_trnslctor ``` | ``` IPR002113 ``` | ``` 0.630518385278751 ``` | ``` 527.743888478315 ``` |
| ``` Ubiquitin ``` | ``` IPR000626 ``` | ``` 0.636268072179031 ``` | ``` 532.556376413849 ``` |
| ``` IF ``` | ``` IPR001664 ``` | ``` 0.637831383447401 ``` | ``` 533.864867945475 ``` |
| ``` Znf_C6HC ``` | ``` IPR002867 ``` | ``` 0.640183412925143 ``` | ``` 535.833516618345 ``` |
| ``` TNFR_c6 ``` | ``` IPR001368 ``` | ``` 0.644889835277926 ``` | ``` 539.772792127624 ``` |
| ``` Zn_MTpeptdse ``` | ``` IPR006025 ``` | ``` 0.652514512547788 ``` | ``` 546.154647002499 ``` |
| ``` Sushi_SCR_CCP ``` | ``` IPR000436 ``` | ``` 0.658293835711165 ``` | ``` 550.991940490245 ``` |
| ``` PMP22_Claudin ``` | ``` IPR004031 ``` | ``` 0.661629089078305 ``` | ``` 553.783547558541 ``` |
| ``` WAP ``` | ``` IPR008197 ``` | ``` 0.662458767518069 ``` | ``` 554.477988412624 ``` |
| ``` RNA_rec_mot ``` | ``` IPR000504 ``` | ``` 0.665260513865187 ``` | ``` 556.823050105162 ``` |
| ``` ER_target ``` | ``` IPR000886 ``` | ``` 0.681640773241132 ``` | ``` 570.533327202828 ``` |
| ``` RasGEFN ``` | ``` IPR000651 ``` | ``` 0.683355918580668 ``` | ``` 571.968903852019 ``` |
| ``` Exo_endo_phos ``` | ``` IPR005135 ``` | ``` 0.689605534995477 ``` | ``` 577.199832791214 ``` |
| ``` UBA_domain ``` | ``` IPR000449 ``` | ``` 0.690596738703965 ``` | ``` 578.029470295219 ``` |
| ``` WD40 ``` | ``` IPR001680 ``` | ``` 0.696368331502875 ``` | ``` 582.860293467906 ``` |
| ``` PCI ``` | ``` IPR000717 ``` | ``` 0.702960087226443 ``` | ``` 588.377593008533 ``` |
| ``` hRIP_like ``` | ``` IPR001164 ``` | ``` 0.704296995334444 ``` | ``` 589.49658509493 ``` |
| ``` Adh_short_C2 ``` | ``` IPR002347 ``` | ``` 0.704522857564751 ``` | ``` 589.685631781697 ``` |
| ``` VWF_C ``` | ``` IPR001007 ``` | ``` 0.717487630038427 ``` | ``` 600.537146342163 ``` |
| ``` 2OG-FeII_Oxy ``` | ``` IPR005123 ``` | ``` 0.72135122802229 ``` | ``` 603.770977854657 ``` |
| ``` C2 ``` | ``` IPR000008 ``` | ``` 0.722599609541164 ``` | ``` 604.815873185954 ``` |
| ``` Znf_RanGDP ``` | ``` IPR001876 ``` | ``` 0.738604354245781 ``` | ``` 618.211844503719 ``` |
| ``` Myb_DNA_binding ``` | ``` IPR001005 ``` | ``` 0.745960762998328 ``` | ``` 624.369158629601 ``` |
| ``` P_rich_extensn ``` | ``` IPR002965 ``` | ``` 0.746103462858882 ``` | ``` 624.488598412884 ``` |
| ``` Glyco_trans_29 ``` | ``` IPR001675 ``` | ``` 0.754789843158659 ``` | ``` 631.759098723798 ``` |
| ``` Neur_channel ``` | ``` IPR006201 ``` | ``` 0.765374376856758 ``` | ``` 640.618353429106 ``` |
| ``` SAP ``` | ``` IPR003034 ``` | ``` 0.769973722211413 ``` | ``` 644.468005490953 ``` |
| ``` Neu_channel_memb ``` | ``` IPR006029 ``` | ``` 0.776186650372567 ``` | ``` 649.668226361838 ``` |
| ``` Srcr_receptor ``` | ``` IPR001190 ``` | ``` 0.784217937881682 ``` | ``` 656.390414006968 ``` |
| ``` PH ``` | ``` IPR001849 ``` | ``` 0.793050160638924 ``` | ``` 663.782984454779 ``` |
| ``` RabGAP_TBC ``` | ``` IPR000195 ``` | ``` 0.796507803257651 ``` | ``` 666.677031326654 ``` |
| ``` snRNP_Sm ``` | ``` IPR001163 ``` | ``` 0.797580607672058 ``` | ``` 667.574968621513 ``` |
| ``` ADH_short ``` | ``` IPR002198 ``` | ``` 0.80826268200054 ``` | ``` 676.515864834452 ``` |
| ``` hormn_receptor ``` | ``` IPR001879 ``` | ``` 0.810116256692725 ``` | ``` 678.067306851811 ``` |
| ``` PDZ ``` | ``` IPR001478 ``` | ``` 0.816547784679674 ``` | ``` 683.450495776887 ``` |
| ``` UIM ``` | ``` IPR003903 ``` | ``` 0.821876039509254 ``` | ``` 687.910245069246 ``` |
| ``` DEAD ``` | ``` IPR001410 ``` | ``` 0.821986315224497 ``` | ``` 688.002545842904 ``` |
| ``` ATP_GTP_A ``` | ``` IPR001687 ``` | ``` 0.82748005112717 ``` | ``` 692.600802793441 ``` |
| ``` Kv_channel ``` | ``` IPR003968 ``` | ``` 0.83290794849674 ``` | ``` 697.143952891771 ``` |
| ``` RasGRF_CDC25 ``` | ``` IPR001895 ``` | ``` 0.843257009101288 ``` | ``` 705.806116617778 ``` |
| ``` Sug_transporter ``` | ``` IPR005829 ``` | ``` 0.847974465774096 ``` | ``` 709.754627852918 ``` |
| ``` PTB_PID ``` | ``` IPR006020 ``` | ``` 0.85296546316002 ``` | ``` 713.932092664937 ``` |
| ``` Sub_transporter ``` | ``` IPR005828 ``` | ``` 0.868996316129788 ``` | ``` 727.349916600633 ``` |
| ``` Znf_CCHC ``` | ``` IPR001878 ``` | ``` 0.870616622512036 ``` | ``` 728.706113042574 ``` |
| ``` GPCR_secretin ``` | ``` IPR000832 ``` | ``` 0.87538734765284 ``` | ``` 732.699209985427 ``` |
| ``` Znf_FYVE ``` | ``` IPR000306 ``` | ``` 0.878631389010476 ``` | ``` 735.414472601768 ``` |
| ``` Sulfotransferase ``` | ``` IPR000863 ``` | ``` 0.88614996487935 ``` | ``` 741.707520604016 ``` |
| ``` CUB_domain ``` | ``` IPR000859 ``` | ``` 0.88731446143537 ``` | ``` 742.682204221405 ``` |
| ``` Znf_ring ``` | ``` IPR001841 ``` | ``` 0.892182779767566 ``` | ``` 746.756986665453 ``` |
| ``` Epsilon_tubulin ``` | ``` IPR004057 ``` | ``` 0.893203051040203 ``` | ``` 747.61095372065 ``` |
| ``` LRR ``` | ``` IPR001611 ``` | ``` 0.897587288026955 ``` | ``` 751.280560078561 ``` |
| ``` Znf_CCCH ``` | ``` IPR000571 ``` | ``` 0.8981751188247 ``` | ``` 751.772574456274 ``` |
| ``` Spectrin ``` | ``` IPR002017 ``` | ``` 0.899819449954917 ``` | ``` 753.148879612266 ``` |
| ``` K+channel_pore ``` | ``` IPR001622 ``` | ``` 0.90602618296812 ``` | ``` 758.343915144316 ``` |
| ``` GST_Cterm ``` | ``` IPR004046 ``` | ``` 0.91184860037595 ``` | ``` 763.21727851467 ``` |
| ``` Cytochrome_P450 ``` | ``` IPR001128 ``` | ``` 0.912649100189898 ``` | ``` 763.887296858945 ``` |
| ``` myosin_head ``` | ``` IPR001609 ``` | ``` 0.917310484720989 ``` | ``` 767.788875711468 ``` |
| ``` RhoGEF ``` | ``` IPR000219 ``` | ``` 0.925174979215054 ``` | ``` 774.371457603 ``` |
| ``` Crystallin ``` | ``` IPR001064 ``` | ``` 0.926110541182021 ``` | ``` 775.154522969352 ``` |
| ``` Pep_M12B_propep ``` | ``` IPR002870 ``` | ``` 0.927240520912175 ``` | ``` 776.10031600349 ``` |
| ``` Thioredox_dom2 ``` | ``` IPR006663 ``` | ``` 0.931749241540606 ``` | ``` 779.874115169487 ``` |
| ``` Reprolysin ``` | ``` IPR001590 ``` | ``` 0.935978731973281 ``` | ``` 783.414198661636 ``` |
| ``` TPR ``` | ``` IPR001440 ``` | ``` 0.936430829028024 ``` | ``` 783.792603896456 ``` |
| ``` Tubulin_FtsZ ``` | ``` IPR003008 ``` | ``` 0.939946275113794 ``` | ``` 786.735032270246 ``` |
| ``` Prenyl_site ``` | ``` IPR001230 ``` | ``` 0.940179693709721 ``` | ``` 786.930403635036 ``` |
| ``` Ig-like ``` | ``` IPR007110 ``` | ``` 0.941166192546547 ``` | ``` 787.75610316146 ``` |
| ``` Lectin_C ``` | ``` IPR001304 ``` | ``` 0.94126571876802 ``` | ``` 787.839406608833 ``` |
| ``` tRNA-synt_I ``` | ``` IPR001412 ``` | ``` 0.943668084767478 ``` | ``` 789.850186950379 ``` |
| ``` SH3 ``` | ``` IPR001452 ``` | ``` 0.946502060010049 ``` | ``` 792.222224228411 ``` |
| ``` kinesin_motor ``` | ``` IPR001752 ``` | ``` 0.947159371736335 ``` | ``` 792.772394143312 ``` |
| ``` Serpin ``` | ``` IPR000215 ``` | ``` 0.953506574064225 ``` | ``` 798.085002491756 ``` |
| ``` WW_Rsp5_WWP ``` | ``` IPR001202 ``` | ``` 0.959091771000595 ``` | ``` 802.759812327498 ``` |
| ``` EP450I ``` | ``` IPR002401 ``` | ``` 0.964006393849449 ``` | ``` 806.873351651989 ``` |
| ``` Ser_estrs_site ``` | ``` IPR000379 ``` | ``` 0.96612726951223 ``` | ``` 808.648524581737 ``` |
| ``` Lipocln_cytFABP ``` | ``` IPR000566 ``` | ``` 0.96623777708194 ``` | ``` 808.741019417584 ``` |
| ``` SH2 ``` | ``` IPR000980 ``` | ``` 0.966660956386563 ``` | ``` 809.095220495553 ``` |
| ``` Collagen ``` | ``` IPR008160 ``` | ``` 0.969585731778981 ``` | ``` 811.543257499007 ``` |
| ``` EF-hand ``` | ``` IPR002048 ``` | ``` 0.970112547545844 ``` | ``` 811.984202295871 ``` |
| ``` Znf_Bbox ``` | ``` IPR000315 ``` | ``` 0.974142847419215 ``` | ``` 815.357563289883 ``` |
| ``` Butyrophylin ``` | ``` IPR003879 ``` | ``` 0.992738383275854 ``` | ``` 830.92202680189 ``` |
| ``` Chymotrypsin ``` | ``` IPR001314 ``` | ``` 0.993139831216944 ``` | ``` 831.258038728582 ``` |
| ``` Ser_protease_Try ``` | ``` IPR001254 ``` | ``` 0.997528712913033 ``` | ``` 834.931532708209 ``` |
| ``` SPRY_receptor ``` | ``` IPR003877 ``` | ``` 0.998227083532244 ``` | ``` 835.516068916488 ``` |
| ``` Hist_TAF ``` | ``` IPR007124 ``` | ``` 0.998793322121151 ``` | ``` 835.990010615403 ``` |
| ``` KRAB ``` | ``` IPR001909 ``` | ``` 0.99999971315991 ``` | ``` 836.999759914845 ``` |
| ``` Znf_C2H2_sub ``` | ``` IPR007086 ``` | ``` 0.999999951606772 ``` | ``` 836.999959494868 ``` |
| ``` GPCR_Rhodpsn ``` | ``` IPR000276 ``` | ``` 0.999999999824772 ``` | ``` 836.999999853334 ``` |
