# Supplementary material for: Arrays of ultraconserved non-coding regions span the loci of key developmental genes in vertebrate genomes
Source: BMC Genomics. 2004 Dec 21;5:99. doi: 10.1186/1471-2164-5-99 (PMC544600; doi:10.1186/1471-2164-5-99)
Supplement: Additional File 4 — Complete list of protein domains in genes with UCR(s) in intron(s) Each tested domain is listed along with corrected and uncorrected P-value as in Table 1. [file 1471-2164-5-99-S4.htm]

Table S3


Complete list of protein
domains in genes with UCR(s) in intron(s)

| ``` domain label ``` | ``` interpro_id ``` | ``` fisher test p value ``` | ``` Bonferroni-corrected p value ``` |
| --- | --- | --- | --- |
| ``` Homeobox ``` | ``` IPR001356 ``` | ``` <1E-18 ``` | ``` ��� <1E-18 ``` |
| ``` Znf_C2H2 ``` | ``` IPR007087 ``` | ``` 4.29514734889835e-09 ``` | ``` 1.57631907704569e-06 ``` |
| ``` Antennapedia ``` | ``` IPR001827 ``` | ``` 2.04348670984622e-07 ``` | ``` 7.49959622513563e-05 ``` |
| ``` HTH_lambrepressr ``` | ``` IPR000047 ``` | ``` 5.73603109343956e-07 ``` | ``` 0.000210512341129232 ``` |
| ``` Paired_box ``` | ``` IPR001523 ``` | ``` 2.06317701356706e-06 ``` | ``` 0.000757185963979111 ``` |
| ``` NLS_BP ``` | ``` IPR001472 ``` | ``` 2.12242647945615e-06 ``` | ``` 0.000778930517960407 ``` |
| ``` MH1 ``` | ``` IPR004863 ``` | ``` 6.12298631796904e-06 ``` | ``` 0.00224713597869464 ``` |
| ``` Homeo_OAR ``` | ``` IPR003654 ``` | ``` 1.11982681347822e-05 ``` | ``` 0.00410976440546507 ``` |
| ``` Laminin_G ``` | ``` IPR001791 ``` | ``` 3.14393609195385e-05 ``` | ``` 0.0115382454574706 ``` |
| ``` IPT_TIG ``` | ``` IPR002909 ``` | ``` 3.34178148064801e-05 ``` | ``` 0.0122643380339782 ``` |
| ``` TF_COE ``` | ``` IPR003523 ``` | ``` 8.47061995115084e-05 ``` | ``` 0.0310871752207236 ``` |
| ``` CTF_NFI_family ``` | ``` IPR000647 ``` | ``` 0.000146428034217716 ``` | ``` 0.0537390885579018 ``` |
| ``` ATP_GTP_A ``` | ``` IPR001687 ``` | ``` 0.00023113207383485 ``` | ``` 0.08482547109739 ``` |
| ``` Hmoeo_CUT ``` | ``` IPR003350 ``` | ``` 0.000231429893071233 ``` | ``` 0.0849347707571425 ``` |
| ``` HMG_12_box ``` | ``` IPR000910 ``` | ``` 0.000252167284175764 ``` | ``` 0.0925453932925054 ``` |
| ``` DEAD_box ``` | ``` IPR000629 ``` | ``` 0.000374083886883314 ``` | ``` 0.137288786486176 ``` |
| ``` CytC_heme_bind ``` | ``` IPR000345 ``` | ``` 0.000407182781338222 ``` | ``` 0.149436080751127 ``` |
| ``` Beige_BEACH ``` | ``` IPR000409 ``` | ``` 0.000483922805454617 ``` | ``` 0.177599669601844 ``` |
| ``` Asx_hydroxyl ``` | ``` IPR000152 ``` | ``` 0.000562865345208219 ``` | ``` 0.206571581691416 ``` |
| ``` TF_Fork_head ``` | ``` IPR001766 ``` | ``` 0.000828886870752443 ``` | ``` 0.304201481566147 ``` |
| ``` EGF_like ``` | ``` IPR006209 ``` | ``` 0.000958596059735028 ``` | ``` 0.351804753922755 ``` |
| ``` IQ_region ``` | ``` IPR000048 ``` | ``` 0.000977226714740542 ``` | ``` 0.358642204309779 ``` |
| ``` PRO_rich ``` | ``` IPR000694 ``` | ``` 0.00141564275192463 ``` | ``` 0.519540889956339 ``` |
| ``` Znf_GATA ``` | ``` IPR000679 ``` | ``` 0.00172619948272601 ``` | ``` 0.633515210160446 ``` |
| ``` Znf_C4steroid ``` | ``` IPR001628 ``` | ``` 0.00174925402273229 ``` | ``` 0.64197622634275 ``` |
| ``` Hormone_rec_lig ``` | ``` IPR000536 ``` | ``` 0.00206464279524787 ``` | ``` 0.757723905855968 ``` |
| ``` Laminin_EGF ``` | ``` IPR002049 ``` | ``` 0.002314228105882 ``` | ``` 0.849321714858694 ``` |
| ``` uDENN ``` | ``` IPR005113 ``` | ``` 0.00251775292393996 ``` | ``` 0.924015323085965 ``` |
| ``` P_rich_extensn ``` | ``` IPR002965 ``` | ``` 0.00342044449137824 ``` | ``` 1.25530312833581 ``` |
| ``` dDENN ``` | ``` IPR005112 ``` | ``` 0.0035016303082287 ``` | ``` 1.28509832311993 ``` |
| ``` Transform_Ski ``` | ``` IPR003380 ``` | ``` 0.00386153056964256 ``` | ``` 1.41718171905882 ``` |
| ``` WD40 ``` | ``` IPR001680 ``` | ``` 0.00402226529745286 ``` | ``` 1.4761713641652 ``` |
| ``` DEAD ``` | ``` IPR001410 ``` | ``` 0.00433617370104999 ``` | ``` 1.59137574828535 ``` |
| ``` M_repeat ``` | ``` IPR003345 ``` | ``` 0.00506677624579344 ``` | ``` 1.85950688220619 ``` |
| ``` Helicase_C ``` | ``` IPR001650 ``` | ``` 0.00528629873325681 ``` | ``` 1.94007163510525 ``` |
| ``` TLE_N ``` | ``` IPR005617 ``` | ``` 0.0053475715964082 ``` | ``` 1.96255877588181 ``` |
| ``` PBX ``` | ``` IPR005542 ``` | ``` 0.00705295216321389 ``` | ``` 2.5884334438995 ``` |
| ``` Highmoblty_12 ``` | ``` IPR000135 ``` | ``` 0.00772873834825383 ``` | ``` 2.83644697380916 ``` |
| ``` NOR1_receptor ``` | ``` IPR003072 ``` | ``` 0.00897010480469151 ``` | ``` 3.29202846332178 ``` |
| ``` SET ``` | ``` IPR001214 ``` | ``` 0.0104269345803590 ``` | ``` 3.82668499099175 ``` |
| ``` R3H ``` | ``` IPR001374 ``` | ``` 0.0110916502527119 ``` | ``` 4.07063564274527 ``` |
| ``` RNA_rec_mot ``` | ``` IPR000504 ``` | ``` 0.0131261149675129 ``` | ``` 4.81728419307723 ``` |
| ``` PH ``` | ``` IPR001849 ``` | ``` 0.0132893993789731 ``` | ``` 4.87720957208313 ``` |
| ``` ZU5 ``` | ``` IPR000906 ``` | ``` 0.0134103936599237 ``` | ``` 4.921614473192 ``` |
| ``` HECT_domain ``` | ``` IPR000569 ``` | ``` 0.0140510181681267 ``` | ``` 5.1567236677025 ``` |
| ``` Cadherin ``` | ``` IPR002126 ``` | ``` 0.0143498470065361 ``` | ``` 5.26639385139875 ``` |
| ``` Ser_thr_pkinase ``` | ``` IPR002290 ``` | ``` 0.0146417890432168 ``` | ``` 5.37353657886057 ``` |
| ``` Ets ``` | ``` IPR000418 ``` | ``` 0.0153194102941321 ``` | ``` 5.62222357794648 ``` |
| ``` PfkB ``` | ``` IPR002173 ``` | ``` 0.0159193200503220 ``` | ``` 5.84239045846817 ``` |
| ``` Clat_adaptor_s ``` | ``` IPR000804 ``` | ``` 0.0186115908243107 ``` | ``` 6.83045383252203 ``` |
| ``` EGF_II ``` | ``` IPR001438 ``` | ``` 0.0210200619765212 ``` | ``` 7.71436274538328 ``` |
| ``` Spectrin_PH ``` | ``` IPR001605 ``` | ``` 0.0214805397437025 ``` | ``` 7.88335808593882 ``` |
| ``` Rtnoid_receptor ``` | ``` IPR003078 ``` | ``` 0.0245196690548040 ``` | ``` 8.99871854311307 ``` |
| ``` EGF_Ca ``` | ``` IPR001881 ``` | ``` 0.0275666338532898 ``` | ``` 10.1169546241574 ``` |
| ``` FN_III ``` | ``` IPR003961 ``` | ``` 0.0303568433826638 ``` | ``` 11.1409615214376 ``` |
| ``` Importinb_N ``` | ``` IPR001494 ``` | ``` 0.0310832981237289 ``` | ``` 11.4075704114085 ``` |
| ``` UPF0171 ``` | ``` IPR005365 ``` | ``` 0.0325697846929686 ``` | ``` 11.9531109823195 ``` |
| ``` Involucrin_rpt ``` | ``` IPR000354 ``` | ``` 0.0345956120774311 ``` | ``` 12.6965896324172 ``` |
| ``` Bromodomain ``` | ``` IPR001487 ``` | ``` 0.035443025456372 ``` | ``` 13.0075903424885 ``` |
| ``` LIM ``` | ``` IPR001781 ``` | ``` 0.0411393622421296 ``` | ``` 15.0981459428616 ``` |
| ``` NMDA_receptor ``` | ``` IPR001508 ``` | ``` 0.042051935350205 ``` | ``` 15.4330602735252 ``` |
| ``` Znf_PHD ``` | ``` IPR001965 ``` | ``` 0.0427657305054825 ``` | ``` 15.6950230955121 ``` |
| ``` FnIII_subd ``` | ``` IPR003962 ``` | ``` 0.0441427460749322 ``` | ``` 16.2003878095001 ``` |
| ``` Transposase_1 ``` | ``` IPR001888 ``` | ``` 0.0484556602858225 ``` | ``` 17.7832273248969 ``` |
| ``` ANK ``` | ``` IPR002110 ``` | ``` 0.050121851392126 ``` | ``` 18.3947194609102 ``` |
| ``` LRR_Nterm ``` | ``` IPR000372 ``` | ``` 0.0512980460596926 ``` | ``` 18.8263829039072 ``` |
| ``` RtnoidX_receptor ``` | ``` IPR000003 ``` | ``` 0.0542322104098694 ``` | ``` 19.9032212204221 ``` |
| ``` UPF0017 ``` | ``` IPR000952 ``` | ``` 0.0640813124329254 ``` | ``` 23.5178416628836 ``` |
| ``` C2 ``` | ``` IPR000008 ``` | ``` 0.0651964689093824 ``` | ``` 23.9271040897433 ``` |
| ``` Mitoch_carrier ``` | ``` IPR001993 ``` | ``` 0.0671418208374348 ``` | ``` 24.6410482473386 ``` |
| ``` TSC-22_Dip_Bun ``` | ``` IPR000580 ``` | ``` 0.0794509932376224 ``` | ``` 29.1585145182074 ``` |
| ``` Prot_kinase ``` | ``` IPR000719 ``` | ``` 0.0833747593419731 ``` | ``` 30.5985366785041 ``` |
| ``` Znf_C2HC ``` | ``` IPR002515 ``` | ``` 0.094568885763968 ``` | ``` 34.7067810753763 ``` |
| ``` Ig_MHC ``` | ``` IPR003006 ``` | ``` 0.0995071294977832 ``` | ``` 36.5191165256864 ``` |
| ``` Death ``` | ``` IPR000488 ``` | ``` 0.101833710596318 ``` | ``` 37.3729717888487 ``` |
| ``` Antifreeze_1 ``` | ``` IPR000104 ``` | ``` 0.107070573873601 ``` | ``` 39.2949006116116 ``` |
| ``` V-SNARE ``` | ``` IPR007705 ``` | ``` 0.109439104801435 ``` | ``` 40.1641514621266 ``` |
| ``` RA_domain ``` | ``` IPR000159 ``` | ``` 0.112377854198252 ``` | ``` 41.2426724907585 ``` |
| ``` Spectrin ``` | ``` IPR002017 ``` | ``` 0.123188929921057 ``` | ``` 45.2103372810279 ``` |
| ``` KID_repeat ``` | ``` IPR003900 ``` | ``` 0.12406569803472 ``` | ``` 45.5321111787422 ``` |
| ``` hRIP_like ``` | ``` IPR001164 ``` | ``` 0.134238474587041 ``` | ``` 49.265520173444 ``` |
| ``` SMC_C ``` | ``` IPR003405 ``` | ``` 0.138452647208695 ``` | ``` 50.8121215255911 ``` |
| ``` Peptidase_S9 ``` | ``` IPR001375 ``` | ``` 0.152603869052674 ``` | ``` 56.0056199423314 ``` |
| ``` Tropomyosin ``` | ``` IPR000533 ``` | ``` 0.156946860483283 ``` | ``` 57.5994977973649 ``` |
| ``` ANF_receptor ``` | ``` IPR001828 ``` | ``` 0.1627327573687 ``` | ``` 59.7229219543129 ``` |
| ``` WWE_dom ``` | ``` IPR004170 ``` | ``` 0.166523216632300 ``` | ``` 61.1140205040541 ``` |
| ``` LRR_Cterm ``` | ``` IPR000483 ``` | ``` 0.176059644789499 ``` | ``` 64.6138896377461 ``` |
| ``` Peptidase_M24 ``` | ``` IPR000994 ``` | ``` 0.180214479945734 ``` | ``` 66.1387141400844 ``` |
| ``` ThyrH_receptor ``` | ``` IPR001728 ``` | ``` 0.193681387338169 ``` | ``` 71.081069153108 ``` |
| ``` Maternal_tudor ``` | ``` IPR008191 ``` | ``` 0.206927606380951 ``` | ``` 75.942431541809 ``` |
| ``` PX ``` | ``` IPR001683 ``` | ``` 0.210151329610012 ``` | ``` 77.1255379668744 ``` |
| ``` PDZ ``` | ``` IPR001478 ``` | ``` 0.215806327487943 ``` | ``` 79.2009221880751 ``` |
| ``` Tyr_PP ``` | ``` IPR000242 ``` | ``` 0.216182351867273 ``` | ``` 79.3389231352892 ``` |
| ``` UBX ``` | ``` IPR001012 ``` | ``` 0.219956744791631 ``` | ``` 80.7241253385286 ``` |
| ``` TYR_phosphatase ``` | ``` IPR000387 ``` | ``` 0.226549262547571 ``` | ``` 83.1435793549585 ``` |
| ``` SPRY_receptor ``` | ``` IPR003877 ``` | ``` 0.230881045067727 ``` | ``` 84.7333435398558 ``` |
| ``` Znf_ZZ ``` | ``` IPR000433 ``` | ``` 0.232772351363856 ``` | ``` 85.4274529505352 ``` |
| ``` HLH_basic ``` | ``` IPR001092 ``` | ``` 0.236732065128752 ``` | ``` 86.880667902252 ``` |
| ``` TF_bZIP ``` | ``` IPR004827 ``` | ``` 0.240438869664212 ``` | ``` 88.2410651667658 ``` |
| ``` SigPTase ``` | ``` IPR000508 ``` | ``` 0.245377917218433 ``` | ``` 90.0536956191649 ``` |
| ``` WH2 ``` | ``` IPR003124 ``` | ``` 0.257776876362188 ``` | ``` 94.604113624923 ``` |
| ``` SHprot_acsite ``` | ``` IPR000169 ``` | ``` 0.264812224748474 ``` | ``` 97.18608648269 ``` |
| ``` Wnt_grthfactor ``` | ``` IPR005816 ``` | ``` 0.269972606817895 ``` | ``` 99.0799467021675 ``` |
| ``` VWF_A ``` | ``` IPR002035 ``` | ``` 0.277006435907433 ``` | ``` 101.661361978028 ``` |
| ``` Sig54_interact ``` | ``` IPR002078 ``` | ``` 0.281968431660369 ``` | ``` 103.482414419355 ``` |
| ``` Aldehyde_dehydr ``` | ``` IPR002086 ``` | ``` 0.283099624126782 ``` | ``` 103.897562054529 ``` |
| ``` TIR_domain ``` | ``` IPR000157 ``` | ``` 0.293767619530476 ``` | ``` 107.812716367685 ``` |
| ``` Prenyl_site ``` | ``` IPR001230 ``` | ``` 0.294934253276328 ``` | ``` 108.240870952412 ``` |
| ``` TGFb_N ``` | ``` IPR001111 ``` | ``` 0.305373386000557 ``` | ``` 112.072032662204 ``` |
| ``` Calponin-like ``` | ``` IPR001715 ``` | ``` 0.313466415116334 ``` | ``` 115.042174347695 ``` |
| ``` Ppantne_attach ``` | ``` IPR006162 ``` | ``` 0.316788893965692 ``` | ``` 116.261524085409 ``` |
| ``` HSF_ETS ``` | ``` IPR002341 ``` | ``` 0.328017254865636 ``` | ``` 120.382332535688 ``` |
| ``` RhoGAP ``` | ``` IPR000198 ``` | ``` 0.331558027773444 ``` | ``` 121.681796192854 ``` |
| ``` Ras_trnsfrmng ``` | ``` IPR001806 ``` | ``` 0.338935860329823 ``` | ``` 124.389460741045 ``` |
| ``` IL1_HBGF ``` | ``` IPR002348 ``` | ``` 0.339061529379582 ``` | ``` 124.435581282307 ``` |
| ``` ABC_transporter ``` | ``` IPR003439 ``` | ``` 0.349512757395211 ``` | ``` 128.271181964042 ``` |
| ``` N6_Mtase ``` | ``` IPR002052 ``` | ``` 0.349924728157377 ``` | ``` 128.422375233757 ``` |
| ``` BTB_POZ ``` | ``` IPR000210 ``` | ``` 0.360060259620296 ``` | ``` 132.142115280649 ``` |
| ``` UIM ``` | ``` IPR003903 ``` | ``` 0.360609812770278 ``` | ``` 132.343801286692 ``` |
| ``` Chromo ``` | ``` IPR000953 ``` | ``` 0.371119696507164 ``` | ``` 136.200928618129 ``` |
| ``` Myb_DNA_binding ``` | ``` IPR001005 ``` | ``` 0.373189496720782 ``` | ``` 136.960545296527 ``` |
| ``` RasGRF_CDC25 ``` | ``` IPR001895 ``` | ``` 0.381457245157926 ``` | ``` 139.994808972959 ``` |
| ``` Ser_estrs_site ``` | ``` IPR000379 ``` | ``` 0.390716755850418 ``` | ``` 143.393049397103 ``` |
| ``` TPR ``` | ``` IPR001440 ``` | ``` 0.391236709158658 ``` | ``` 143.583872261227 ``` |
| ``` PTB_PID ``` | ``` IPR006020 ``` | ``` 0.391625277607172 ``` | ``` 143.726476881832 ``` |
| ``` DS_phosphatase ``` | ``` IPR000340 ``` | ``` 0.401626566868362 ``` | ``` 147.396950040689 ``` |
| ``` Znf_CCHC ``` | ``` IPR001878 ``` | ``` 0.411463840702397 ``` | ``` 151.00722953778 ``` |
| ``` Znf_FYVE ``` | ``` IPR000306 ``` | ``` 0.421139782253503 ``` | ``` 154.558300087036 ``` |
| ``` KH_dom ``` | ``` IPR004087 ``` | ``` 0.430657030996621 ``` | ``` 158.05113037576 ``` |
| ``` Crystallin ``` | ``` IPR001064 ``` | ``` 0.43631692071689 ``` | ``` 160.128309903099 ``` |
| ``` Zn_carbOpept ``` | ``` IPR000834 ``` | ``` 0.440018183262878 ``` | ``` 161.486673257476 ``` |
| ``` cNMP_binding ``` | ``` IPR000595 ``` | ``` 0.458282372541127 ``` | ``` 168.189630722594 ``` |
| ``` kazal ``` | ``` IPR002350 ``` | ``` 0.467190393109272 ``` | ``` 171.458874271103 ``` |
| ``` myosin_head ``` | ``` IPR001609 ``` | ``` 0.475952285719474 ``` | ``` 174.674488859047 ``` |
| ``` LDL_receptor_A ``` | ``` IPR002172 ``` | ``` 0.501384913575588 ``` | ``` 184.008263282241 ``` |
| ``` UBQ_conjugat ``` | ``` IPR000608 ``` | ``` 0.509585819619653 ``` | ``` 187.017995800413 ``` |
| ``` Reg_chr_condens ``` | ``` IPR000408 ``` | ``` 0.525586166763524 ``` | ``` 192.890123202213 ``` |
| ``` Hydrolase ``` | ``` IPR005834 ``` | ``` 0.533389976612234 ``` | ``` 195.75412141669 ``` |
| ``` Serpin ``` | ``` IPR000215 ``` | ``` 0.548615524500933 ``` | ``` 201.341897491842 ``` |
| ``` WW_Rsp5_WWP ``` | ``` IPR001202 ``` | ``` 0.563345447378433 ``` | ``` 206.747779187885 ``` |
| ``` EP450I ``` | ``` IPR002401 ``` | ``` 0.577595840288456 ``` | ``` 211.977673385863 ``` |
| ``` Lipocln_cytFABP ``` | ``` IPR000566 ``` | ``` 0.584546099670596 ``` | ``` 214.528418579109 ``` |
| ``` Ion_trans ``` | ``` IPR005821 ``` | ``` 0.588752747062782 ``` | ``` 216.072258172041 ``` |
| ``` ER_target ``` | ``` IPR000886 ``` | ``` 0.591382276879479 ``` | ``` 217.037295614769 ``` |
| ``` Adh_short_C2 ``` | ``` IPR002347 ``` | ``` 0.604719826205454 ``` | ``` 221.932176217402 ``` |
| ``` EF-hand ``` | ``` IPR002048 ``` | ``` 0.620374211531576 ``` | ``` 227.677335632088 ``` |
| ``` DAG_PE-bind ``` | ``` IPR002219 ``` | ``` 0.630106113796735 ``` | ``` 231.248943763402 ``` |
| ``` SH3 ``` | ``` IPR001452 ``` | ``` 0.647167893361678 ``` | ``` 237.510616863736 ``` |
| ``` Cytochrome_P450 ``` | ``` IPR001128 ``` | ``` 0.648072549835539 ``` | ``` 237.842625789643 ``` |
| ``` Ig-like ``` | ``` IPR007110 ``` | ``` 0.64830207500816 ``` | ``` 237.926861527995 ``` |
| ``` Ubiquitin ``` | ``` IPR000626 ``` | ``` 0.670680844381251 ``` | ``` 246.139869887919 ``` |
| ``` Exo_endo_phos ``` | ``` IPR005135 ``` | ``` 0.696914502190058 ``` | ``` 255.767622303751 ``` |
| ``` Butyrophylin ``` | ``` IPR003879 ``` | ``` 0.721063126555374 ``` | ``` 264.630167445822 ``` |
| ``` Collagen ``` | ``` IPR008160 ``` | ``` 0.747521096990207 ``` | ``` 274.340242595406 ``` |
| ``` LRR ``` | ``` IPR001611 ``` | ``` 0.749369020688615 ``` | ``` 275.018430592722 ``` |
| ``` Znf_Bbox ``` | ``` IPR000315 ``` | ``` 0.759795752331336 ``` | ``` 278.8450411056 ``` |
| ``` Hist_TAF ``` | ``` IPR007124 ``` | ``` 0.824838957478084 ``` | ``` 302.715897394457 ``` |
| ``` Tyr_pkinase ``` | ``` IPR001245 ``` | ``` 0.906914907711119 ``` | ``` 332.837771129981 ``` |
| ``` Znf_ring ``` | ``` IPR001841 ``` | ``` 0.988644422375584 ``` | ``` 362.832503011839 ``` |
| ``` GPCR_Rhodpsn ``` | ``` IPR000276 ``` | ``` 0.999998287288042 ``` | ``` 366.999371434711 ``` |
