# Supplementary material for: Arrays of ultraconserved non-coding regions span the loci of key developmental genes in vertebrate genomes
Source: BMC Genomics. 2004 Dec 21;5:99. doi: 10.1186/1471-2164-5-99 (PMC544600; doi:10.1186/1471-2164-5-99)
Supplement: Additional File 6 — Genes associated with enumerated UCR clusters from Figure 3. UCRs were counted by sliding a 500 kb window along the chromosomes. Overlapping UCR-containing windows were merged into a single cluster span. The cluster span coordinates correspond to the human genome NCBI build 33 (UCSC hg15, April 2003). A more exhaustive list is found in [Additional file 7] [file 1471-2164-5-99-S6.htm]

Nr


|  |  |  |  |  |  |  |
| --- | --- | --- | --- | --- | --- | --- |
| ***Nr.*** | ***UCR cluster span*** | ***Nr UCRs*** | ***Gene Symbol*** | ***Gene Description*** | ***InterPro domain(s)*** | ***Evidence for involvement �in embryonic development?***  ***(PubMed reference in brackets)*** |
| 1. | chr15:32.7M-36.2M | 84 | **MEIS2** | Meis1, myeloid ecotropic viral integration site 1 homolog 2� | Homeobox | YES [12880965] |
| 2. | chr2:143.5M-147.8M | 81 | **ZFHX1B** | zinc finger homeobox 1b | Homeobox  Zn-finger, C2H2 type | YES [15006694] |
| 3. | chr19:34.8M-37.8M | 80 | **ZNF536** | KIAA0390 gene product (zinc finger) | Znf\_C2H2, NLS\_BP | YES [10819330] |
| 4. | chr10:129.6M-132.1M | 79 | **EBF3** | COE3 (Early B-cell factor 3, EBF-3) |  | YES [14732407] |
| 5. | chr10:76.5M-79.1M | 77 | **ZNF503** | zinc finger protein 503 | Znf\_PHD  Znf\_C2H2 Eggshell | YES [14983057] |
| 6. | chr16:52.9M-55.6M | 64 | **IRX-3**  **IRX-5**  **IRX-6** | Iroquis-class protein IRX-3   Iroquis-class protein IRX-5   Iroquis-class protein IRX-6 | Homeobox  Homeobox  Homeobox | YES [12100884] |
| 7. | chr9:121.2M-123.3M | 62 | **PBX3** | pre-B-cell leukemia transcription factor 3 | PBX  Homeobox | YES [15466398] |
| 8. | chr5:90.6M-94.4M | 62 | **NR2F1** | nuclear receptor subfamily 2, group F, member 1 | Hormone\_rec\_lig  Stdhrmn\_receptor  Str\_ncl\_receptor  Znf\_C4steroid | YES [11784326] |
| 9. | chr7:112.9M-115.4M | 60 | **FOXP2 ;  TFEC** | forkhead box P2 (immune tolerance development) ;  Similar to transcription factor EC | Involucrin\_rpt TF\_Fork\_head Znf\_C2H2   -------  HLH\_basic | YES [12876151] |
| 10. | chr13:70.2M-72.5M | 52 | **DACH** | dachshund homolog (Drosophila) | Transform\_Ski | YES [10767315] |
| 11. | chr10:102.1M-104.6M | 52 | **PAX2** | paired box gene 2 (kidney, differentiation, eyes, CNS) | Paired\_box Homeobox | YES [15242798] |
| 12. | chr3:69.5M-71.7M | 52 | **FOXP1** | forkhead box P1 | TF\_Fork\_head  Znf\_C2H2 | YES [12687690] |
| 13. | chr2:58.3M-61.2M | 48 | **BCL11A** | B-cell lymphoma/leukemia 11A (COUP-TF interacting protein 1) | Znf\_C2H2 | YES [12711548] |
| 14. | chr5:1.7M-5.1M | 46 | **IRX-4;**  **IRX-2;**  **IRX-1** | Homeobox protein IRX-4;  Homeobox protein IRX-2;  Homeobox protein IRX-1 | Homeobox    Homeobox    Homeobox | YES  [11335133], [11455441] |
| 15. | chr2:175.7M-178.0M | 46 | **ATF-2;   EVX-2;   HOX-D\*** | activating transcription factor 2 (brain) ;  Homeobox even-skipped homolog protein 2 (EVX-2) ;  HOX-D cluster | Znf\_C2H2 TF\_bZIP  --------------------  Homeobox Antifreeze\_1 HTH\_lambrepressr CytC\_heme\_bind  --------------------  Homeobox HTH\_lambrepressr | YES [8978698] |
| 16. | chr2:155.9M-158.3M | 41 | **NR4A2** | nuclear receptor subfamily 4, group A, member 2 (brain) | Znf\_C4steroid   Hormone\_rec\_lig   NURR\_receptor   Nucorph\_receptor   Stdhrmn\_receptor   Rtnoid\_receptor   VitD\_receptor | YES [15009684] |
| 17. | chr1:62.3M-63.6M | 39 | **FOXD3** | forkhead, box D3 | TF\_Fork\_head | YES [11262245] |
| 18. | chr1:89.5M-91.1M | 38 | **LMO4 ;**  **KIAA1221** | LIM domain only 4;  KIAA1221 (brain-specific zinc finger protein) | LIM  -----------  Znf\_C2H2 | YES [12619132] |
| 19. | chr18:71.7M-74.1M | 38 | **ZNF407** | zinc finger protein 407 | Znf\_C2H2 | NO |
| 20. | chr2:65.6M-67.9M | 35 | **MEIS1** | Meis1, myeloid ecotropic viral integration site 1 homolog (mouse) | Homeobox | YES [12183364] |
| 21. | chr8:105.2M-106.9M | 35 | **ZFPM2** | zinc finger protein, multitype 2 ((FOG-2)� (Friend of GATA-2) | Znf\_C2H2 | YES [12213678] |
| 22. | chr16:50.2M-52.9M | 35 | **TNRC9** | trinucleotide repeat containing 9 (HMG box DNA binding protein) | Highmoblty\_12  HMG-box  HMG\_12\_box | NO |
| 23. | chr8:76.2M-78.3M | 33 | **ZFH4** | zinc finger homeodomain 4 | AMP-bind  Homeobox  Somatotropin  Znf\_C2H2  Znf\_U1 | NO |
| 24. | chr11:14.9M-17.0M | 32 | **SOX6** | SRY (sex determining region Y)-box 6 | HMG\_12\_box ATP\_GTP\_A NLS\_BP | YES [12571105] |
| 25. | chr9:15.5M-17.8M | 31 | **BNC2** | Basonuclin 2 | CytC\_heme\_BS  Znf\_C2H2 | NO |
| 26. | chr5:765M-779M | 31 | **OTP** | orthopedia homolog | Homeobox Homeo\_OAR HTH\_lambrepressr | YES [11071765] |
| 27. | chr10:114.0M-115.5M | 30 | **TCF7L2** | transcription factor 7-like 2 (T-cell specific, HMG-box) | HMG-box | YES [11845287] |
| 28. | chr18:74.9M-76.9M | 30 | **SALL3** | Sal-like protein 3 (Zinc finger protein SALL3) (hSALL3) | Znf\_C2H2 | YES [10610715] |
| 29. | chr10:124.4M-125.7M | 27 | **BUB3** | Mitotic checkpoint protein BUB3 | WD40 | YES [10995385] |
| 30. | chr6:8.1M-10.9M | 26 | **TFAP2A** | Transcription factor AP-2 alpha (AP2-alpha) | TF\_AP2  TF\_AP2\_alpha | YES [14534133] |
| 31. | chr4:84.6M-86.2M | 26 | **NKX6-1** | Homeobox protein NKX-6.1 | Homeobox HTH\_lambrepressr PRO\_rich | YES [15456722] |
| 32. | chr1:9.8M-11.0M | 25 | **FLJ20321** | Hypothetical protein FLJ20321 (zinc finger) | Znf\_C2H2   ATP\_GTP\_A   NLS\_BP | NO |
| 33. | chr8:64.5M-66.4M | 25 | **BHLHB5** | Basic helix-loop-helix domain containing, class B, 5 | HLH\_basic | YES [14643684] |
| 34. | chr20:20.4M-21.9M | 25 | **NKX-2.2** | Homeobox protein NKX-2.2 | Homeobox  -------------  Paired\_box | YES [8575293] |
| 35. | chr16:71.5M-74.1M | 25 | **ATBF1** | Alpha-fetoprotein enhancer binding protein (AT motif-binding factor) | Homeobox  Znf\_C2H2 | YES [12926016] |
| 36. | chr1:86.6M-88.5M | 22 | **LMO4** | LIM domain transcription factor LMO4 (LIM-only protein 4) (LMO-4)� | LIM | YES �[12619132] |
| 37. | chr7:68.5M-69.9M | 22 | **AUTS2** | Autism susceptibility candidate (unknown function) | P\_rich\_extensn   NLS\_BP | YES [12160723] |
| 38. | chr6:97.3M-100.5M | 22 | **POU3F2** | Nervous-system specific octamer-binding transcr. factor N-Oct-3 | Homeobox  POU\_domain  POU\_homeo | YES [10064882] |
| 39. | chr10:118.4M-120.8M | 21 | **EMX2** | Homeobox protein EMX2 (empty spiracles homolog 2 (Drosophila)) | Homeobox | YES [15501221] |
| 40. | chr18:22.1M-23.2M | 21 | **EHZF** | Early hematopoietic zinc finger (LYST-interacting protein LIP3) | Znf\_C2H2 | NO |
| 41. | chr11:7.8M-8.8M | 20 | **LMO1** | LIM domain only 1 (rhombotin 1) (expressed mainly in CNS) | LIM | YES [1507224] |
| 42. | chr3:158.6M-159.9M | 20 | **SHOX2** | Short stature homeobox protein 2 (Homeobox protein Og12X) | Homeobox  Homeo\_OAR  HTH\_lambrepressr  Paired\_homeo | YES [14769946] |
| 43. | chr4:111.5M-113.5M | 19 | **PITX2** | Pituitary homeobox 2 (ALL1 responsive protein ARP1) | Homeo\_OAR  Homeobox  Paired\_homeo | YES [14975719] |
| 44. | chr4:79.9M-81.2M | 18 |  | *NOT DETERMINED* |  | NO |
| 45. | chr2:173.8M-175.4M | 18 | **SP3** | Transcription factor Sp3 (SPR-2). | Znf\_C2H2 | NO |
| 46. | chr15:64.3M-66.1M | 18 | **MADH3** | Mothers against decapentaplegic homolog 3 (SMAD 3) | Dwarfin  Dwarfin\_A  SMAD\_FHA | YES [15183723] |
| 47. | chr9:91.1M-92.2M | 18 | **PTCH** | Patched protein homolog 1 (PTC1) | Patched family  Patched transmembrane receptor  Sterol-sensing 5TM box | YES [12917290] |
| 48. | chr4:147.2M-148.9M | 18 | **POU4F2** | POU domain, class 4, transcription factor 2� (Brn-3B). | Homeobox  POU\_domain  POU\_homeo | YES [14973295] |
| 49. | chr2:164.0M-165.4M | 17 | **-** | weakly similar to GC-rich sequence DNA-binding factor (GCF) [Homo sapiens] |  | NO |
| 50. | chr17:34.3M-35.7M | 17 | **LHX1** | LIM homeobox 1 | Homeobox  LIM  LIM\_homeo | YES [12799141] |
