# Supplementary material for: Arrays of ultraconserved non-coding regions span the loci of key developmental genes in vertebrate genomes
Source: BMC Genomics. 2004 Dec 21;5:99. doi: 10.1186/1471-2164-5-99 (PMC544600; doi:10.1186/1471-2164-5-99)
Supplement: Additional File 7 — Extended list of UCR clusters An extended, but less annotated, version of in [Additional file 6] [file 1471-2164-5-99-S7.htm]

chr15:32700001-36200001


An expanded version of Additional
file 6.

|  |  |  |  |  |  |
| --- | --- | --- | --- | --- | --- |
| ***Nr.*** | ***UCR cluster span*** | ***Nr UCRs*** | ***Gene Symbol*** | ***Description*** | ***Interpro domains*** |
|  | chr15:32700001-36200001 | 84 | MEIS2 | Meis1, myeloid ecotropic viral integration site 1 homolog 2 (mouse) | Homeobox |
|  | chr2:143500001-147800001 | 81 | ZFHX1B | zinc finger homeobox 1b | Homeobox  Zn-finger, C2H2 type |
|  | chr19:34800001-37800001 | 80 | KIAA0390 | KIAA0390 gene product | Znf\_C2H2, NLS\_BP |
|  | chr10:129600001-132100001 | 79 | EBF-3 | COE3\_HUMAN , Transcription factor COE3 (Early B-cell factor 3) (EBF-3) |  |
|  | chr10:76500001-79100001 | 77 | ZNF503 | zinc finger protein 503 | Znf\_PHD  Znf\_C2H2 Eggshell |
|  | chr16:52900001-55600001 | 64 | IRX-3  IRX-5  IRX-6 | Iroquis-class protein IRX-3   Iroquis-class protein IRX-5   Iroquis-class protein IRX-6 | Homeobox  Homeobox  Homeobox |
|  | chr9:121200001-123300001 | 62 | PBX3 | pre-B-cell leukemia transcription factor 3 | PBX  Homeobox |
|  | chr5:90600001-94400001 | 62 | NR2F1 | nuclear receptor subfamily 2, group F, member 1 | Hormone\_rec\_lig  Stdhrmn\_receptor  Str\_ncl\_receptor  Znf\_C4steroid |
|  | chr7:112900001-115400001 | 60 | FOXP2   -------  TFEC | forkhead box P2 (immune tolerance development)   -----  Similar to transcription factor EC | Involucrin\_rpt TF\_Fork\_head Znf\_C2H2   -------  HLH\_basic |
|  | chr13:70200001-72500001 | 52 | DACH | dachshund homolog (Drosophila) | Transform\_Ski |
|  | chr10:102100001-104600001 | 52 | PAX2 | paired box gene 2 (kidney, differentiation, eyes, CNS) | Paired\_box Homeobox |
|  | chr3:69500001-71700001 | 52 | FOXP1 | forkhead box P1  (specification and differentiation of lung epithelium) | TF\_Fork\_head Znf\_C2H2 |
|  | chr2:58300001-61200001 | 48 | BCL11A | B-cell lymphoma/leukemia 11A (B-cell CLL/lymphoma 11A) (COUP-TF interacting protein 1) (Ecotropic viral integration site 9 protein) (EVI-9) | Znf\_C2H2 |
|  | chr5:1700001-5100001 | 46 | IRX-4  IRX-2  IRX-1 | IRX-4   IRX-2   IRX-1 | Homeobox  Homeobox  Homeobox |
|  | chr2:175700001-178000001 | 46 | ATF-2  --------- EVX-2  -------  HOX-D\* | activating transcription factor 2 (brain)   -------  HOMEOBOX EVEN-SKIPPED HOMOLOG PROTEIN 2 (EVX-2)   -------  HOX-D cluster | Znf\_C2H2 TF\_bZIP  --------------------  Homeobox Antifreeze\_1 HTH\_lambrepressr CytC\_heme\_bind  --------------------  Homeobox HTH\_lambrepressr |
|  | chr2:155900001-158300001 | 41 | NR4A2 | nuclear receptor subfamily 4, group A, member 2 (brain) | Znf\_C4steroid   Hormone\_rec\_lig   NURR\_receptor   Nucorph\_receptor   Stdhrmn\_receptor   Rtnoid\_receptor   VitD\_receptor |
|  | chr1:62300001-63600001 | 39 | FOXD3 | forkhead, box D3, at chr1:63146833-63147169 | TF\_Fork\_head |
|  | chr1:89500001-91100001 | 38 | LMO4  ----------  KIAA1221 | LIM domain only 4  -----------  KIAA1221 (brain) | LIM  -----------  Znf\_C2H2 |
|  | chr18:71700001-74100001 | 38 | ZNF407 | zinc finger protein 407 | Znf\_C2H2 |
|  | chr2:65600001-67900001 | 35 | MEIS1 | Meis1, myeloid ecotropic viral integration site 1 homolog (mouse) | Homeobox |
|  | chr8:105200001-106900001 | 35 | ZFPM2  (FOG-2) | zinc finger protein, multitype 2 (Friend of GATA-2) (cardiogenesis, hematopoiesis) | Znf\_C2H2 |
|  | chr16:50200001-52900001 | 35 | TNRC9 | trinucleotide repeat containing 9 | Highmoblty\_12  HMG-box  HMG\_12\_box |
|  | chr8:76200001-78300001 | 33 | ZFH4 | zinc finger homeodomain 4 | AMP-bind  Homeobox  Somatotropin  Znf\_C2H2  Znf\_U1 |
|  | chr11:14900001-17000001 | 32 | SOX6 | SRY (sex determining region Y)-box 6 | HMG\_12\_box ATP\_GTP\_A NLS\_BP |
|  | chr9:15500001-17800001 | 31 | FLJ20043 | Hypothetical protein FLJ20043 | CytC\_heme\_BS  Znf\_C2H2 |
|  | chr5:76500001-77900001 | 31 | OTP | orthopedia homolog (development of the neuroendocrine hypothalamus) | Homeobox Homeo\_OAR HTH\_lambrepressr |
|  | chr10:114000001-115500001 | 30 | TCF7L2 | transcription factor 7-like 2 (T-cell specific, HMG-box) |  |
|  | chr18:74900001-76900001 | 30 | SALL3 | Sal-like protein 3 (Zinc finger protein SALL3) (hSALL3) | Znf\_C2H2 |
|  | chr10:124400001-125700001 | 27 | BUB3 | Mitotic checkpoint protein BUB3 | WD40 |
|  | chr6:8100001-10900001 | 26 | TFAP2A | Transcription factor AP-2 alpha (AP2-alpha) (Activating enhancer- binding protein 2 alpha) (AP-2 transcription factor) (Activator protein-2) (AP-2). | TF\_AP2  TF\_AP2\_alpha |
|  | chr4:84600001-86200001 | 26 | NKX6-1 | Homeobox protein NKX-6.1 | Homeobox HTH\_lambrepressr PRO\_rich |
|  | chr1:9800001-11000001 | 25 | FLJ20321 | Hypothetical protein FLJ20321 | Znf\_C2H2   ATP\_GTP\_A   NLS\_BP |
|  | chr8:64500001-66400001 | 25 | BHLHB5 | Basic helix-loop-helix domain containing, class B, 5 | HLH\_basic |
|  | chr20:20400001-21900001 | 25 | NKX-2.2  -----------  PAX-1 | Homeobox protein NKX-2.2 | Homeobox  -------------  Paired\_box |
|  | chr16:71500001-74100001 | 25 | ATBF1 | Alpha-fetoprotein enhancer binding protein (AT motif-binding factor) (AT-binding transcription factor 1) | Homeobox  Znf\_C2H2 |
|  | chr1:86600001-88500001 | 22 | LMO4 | LIM domain transcription factor LMO4 (LIM-only protein 4) (LMO-4) (Breast tumor autoantigen). | LIM |
|  | chr7:68500001-69900001 | 22 | AUTS2 | Autism susceptibility candidate (unknown function) | P\_rich\_extensn   NLS\_BP |
|  | chr6:97300001-100500001 | 22 | POU3F2 | POU domain, class 3, transcription factor 2 (Nervous-system specific octamer-binding transcription factor N-Oct-3) (Brain-specific homeobox/POU domain protein 2) (Brain-2) (Brn-2 protein) | Homeobox  POU\_domain  POU\_homeo |
|  | chr10:118400001-120800001 | 21 | EMX2 | Homeobox protein EMX2 (empty spiracles homolog 2 (Drosophila)) | Homeobox |
|  | chr18:22100001-23200001 | 21 | EHZF | Early hematopoietic zinc finger (LYST-interacting protein LIP3) | Znf\_C2H2 |
|  | chr11:7800001-8800001 | 20 | LMO1 | LIM domain only 1 (rhombotin 1) (expressed mainly in CNS) | LIM |
|  | chr3:158600001-159900001 | 20 | SHOX2 | Short stature homeobox protein 2 (Paired-related homeobox protein SHOT) (Homeobox protein Og12X) | Homeobox  Homeo\_OAR  HTH\_lambrepressr  Paired\_homeo |
|  | chr4:111500001-113500001 | 19 | PITX2 | Pituitary homeobox 2 (RIEG bicoid-related homeobox transcription factor) (Solurshin) (ALL1 responsive protein ARP1) | Homeo\_OAR  Homeobox  Paired\_homeo |
|  | chr4:79900001-81200001 | 18 |  | *NOT DETERMINED* |  |
|  | chr2:173800001-175400001 | 18 | SP3 | Transcription factor Sp3 (SPR-2). | Znf\_C2H2 |
|  | chr15:64300001-66100001 | 18 | MADH3 | Mothers against decapentaplegic homolog 3 (SMAD 3) (Mothers against DPP homolog 3) (Mad3) (hMAD-3) | Dwarfin  Dwarfin\_A  SMAD\_FHA |
|  | chr9:91100001-92200001 | 18 | PTCH | Patched protein homolog 1 (PTC1) | Patched family  Patched transmembrane receptor  Sterol-sensing 5TM box |
|  | chr4:147200001-148900001 | 18 | POU4F2 | POU domain, class 4, transcription factor 2 (Brain-specific homeobox/POU domain protein 3B) (Brn-3B). | Homeobox  POU\_domain  POU\_homeo |
|  | chr2:164000001-165400001 | 17 | - | weakly similar to GC-RICH SEQUENCE DNA-BINDING FACTOR (GCF) (TRANSCRIPTION FACTOR 9) (TCF-9) [Homo sapiens] |  |
|  | chr17:34300001-35700001 | 17 | LHX1 | LIM homeobox 1 | Homeobox  LIM  LIM\_homeo |
|  | chr7:500001-1500001 | 17 | UNC4-1 | UNC4 1 HOMEOBOX C ELEGANS | Homeobox  NLS\_BP |
|  | chr8:36400001-37800001 | 16 | FLJ14299 | Hypothetical protein FLJ14299 | Znf\_C2H2 |
|  | chr1:212400001-213800001 | 16 | ESRRG | Estrogen-related receptor gamma | Hormone\_rec\_lig  Stdhrmn\_receptor  Str\_ncl\_receptor  Znf\_C4steroid |
|  | chr14:95000001-98100001 | 16 | BCL11B | B-cell lymphoma/leukemia 11B (B-cell CLL/lymphoma 11B) (Radiation- induced tumor suppressor gene 1 protein) (hRit1) (COUP-TF interacting protein 2) | Znf\_C2H2 |
|  | chr3:17400001-19100001 | 15 | SATB1 | special AT-rich sequence binding protein 1 | Homeobox |
|  | chr7:26000001-27400001 | 15 | HOXA\* | HOX A cluster | Homeobox |
|  | chr15:57400001-58800001 | 15 | FOXB1 | Forkhead box protein B1 (Transcription factor FKH-5) | TF\_Fork\_head |
|  | chr14:34400001-35900001 | 15 | TITF1 | Thyroid transcription factor 1 (Thyroid nuclear factor 1) (TTF-1) (Homeobox protein Nkx-2.1) (Homeobox protein NK-2 homolog A) | Homeobox |
|  | chr1:37600001-39200001 | 15 | POU3F | POU domain, class 3, transcription factor 1 (Octamer-binding transcription factor 6) (Oct-6) (POU-domain transcription factor SCIP) | POU\_domain  Homeobox |
|  | chr16:77700001-79100001 | 15 | MAF | Transcription factor Maf (Proto-oncogene c-maf) | Euk\_transcr\_DNA  TF\_bZIP  TF\_Maf |
|  | chr7:20200001-21200001 | 15 | SP8  -----  SP4 | Transcription factor Sp8  ------  Transcription factor Sp4 | Znf\_C2H2  -----  Znf\_C2H2 |
|  | chr3:147800001-149500001 | 14 | ZIC4  -----  ZIC1 | Zinc finger protein ZIC 4 (Zinc finger protein of the cerebellum 4)   ----  Zinc finger protein ZIC 1 (Zinc finger protein of the cerebellum 1) | Znf\_C2H2  ------  Znf\_C2H2 |
|  | chr13:98900001-100400001 | 14 | ZIC5  ------  ZIC2 | Zinc finger protein ZIC 5 (Zinc finger protein of the cerebellum 5) (odd-paired homolog (Drosophila))  ------  Zinc finger protein ZIC 2 (Zinc finger protein of the cerebellum 5) (odd-paired homolog (Drosophila)) | Znf\_C2H2  -----  Znf\_C2H2 |
|  | chr4:41200001-42200001 | 14 | PHOX2B | Paired mesoderm homeobox protein 2B (Paired-like homeobox 2B) (PHOX2B homeodomain protein) (Neuroblastoma Phox) (NBPhox) | Homeobox  Paired\_homeo |
|  | chr14:54800001-55900001 | 14 | OTX2 | Orthodenticle 2 | Homeobox  Otx |
|  | chr1:214000001-215300001 | 14 |  | Close to TGFB2, could be an extension of ESRRG SCR cluster, or an uncharacterized nuclear protein CGI-115 |  |
|  | chr18:52200001-53300001 | 13 | TCF4 | transcription factor 4 | HLH\_basic |
|  | chr4:151500001-152500001 | 13 | MAB21L2 | (MAB21L2 protein) (MAB-21 (C. ELEGANS)-like 2 (neurogenesis) | Mab-21  Ricin\_B\_lectin |
|  | chr12:53600001-54600001 | 13 | HOXC\* | Hox-C cluster | Homeobox |
|  | chr15:67400001-68700001 | 13 | TLE3 | Transducin-like enhancer of split 3 (E(sp1) homolog, Drosophila) | TLE\_N  WD40 |
|  | chr3:137800001-139200001 | 12 | SOX14 | Transcription factor SOX-14 | Highmoblty\_12  HMG\_12\_box |
|  | chr13:26600001-27800001 | 12 |  |  |  |
|  | chr5:87500001-88500001 | 12 | MEF2 | MADS box transcription enhancer factor 2, polypeptide C (myocyte enhancer factor 2C) | TF\_MADSbox |
|  | chr13:93700001-94800001 | 12 |  |  |  |
|  | chr14:74100001-75500001 | 12 | ESRRB | Steroid hormone receptor ERR2 (Estrogen-related receptor, beta) (Estrogen receptor-like 2) (ERR beta-2) | Hormone\_rec\_lig  Stdhrmn\_receptor  Str\_ncl\_receptor  Znf\_C4steroid |
|  | chr20:51800001-53100001 | 12 | C20orf17 | Highly similar to Mus musculus teashirt 2 (Tsh2) gene | Homeobox  Znf\_C2H2 |
|  | chr5:157700001-158800001 | 12 | EBF | Transcription factor COE1 (OE-1) (O/E-1) (Early B-cell factor) | HLH\_basic  Ig-like  IPT\_TIG  TF\_COE |
|  | chr1:210200001-211300001 | 12 | PROX1 | Homeobox prospero-like protein PROX1 (PROX 1). | Prox1 |
|  | chr13:77800001-79000001 | 12 | POU4F1 | POU domain, class 4, transcription factor 1 (Brain-specific homeobox/POU domain protein 3A) (Brn-3A) (Oct-T1) | Homeobox  POU\_domain  POU\_homeo |
|  | chr3:169900001-171100001 | 11 | EVI1 | Ecotropic virus integration 1 site protein | Znf\_C2H2 |
|  | chr15:95000001-96300001 | 11 | NR2F2 (perifery of SCR cluster) | nuclear receptor subfamily 2, group F, member 2 (COUP transcription factor 2) | Znf\_C4steroid  Hormone\_rec\_lig  COUP\_TF  Stdhrmn\_receptor  RtnoidX\_receptor  VitD\_receptor  ThyrH\_receptor |
|  | chr17:46400001-47400001 | 11 | HOXB\* | Hox-B cluster | Homeobox |
|  | chr11:31300001-32300001 | 11 | PAX6 | Paired box protein Pax-6 (Oculorhombin) (Aniridia, type II protein). | Homeobox  Paired\_box  Paired\_homeo |
|  | chr3:181900001-183300001 | 10 | SOX2 | Transcription factor SOX-2 | HMG\_12\_box |
|  | chr2:62700001-63700001 | 10 | OTX1 | orthodenticle homolog 1 (Drosophila) | Homeobox  Otx\_TF  Paired\_homeo |
|  | chr2:236300001-237500001 | 10 | GBX2 | Homeobox protein GBX-2 (Gastrulation and brain-specific homeobox protein 2). | Homeobox |
|  | chr7:38500001-39600001 | 10 | RPF-1 | Retina-derived POU-domain factor-1 (RPF-1). | Homeobox  POU\_domain  POU\_homeo |
|  | chrX:23700001-24700001 | 10 | ARX | Homeobox protein ARX (Aristaless related homeobox) | Homeobox  Homeo\_OAR  Paired\_homeo |
|  | chr2:44500001-45500001 | 10 | SIX3  -----  SIX2 | sine oculis homeobox homolog 3 (Drosophila)  -----------  sine oculis homeobox homolog 2 (Drosophila) | Homeobox  HTH\_lambrepressr  SIX  SIX\_SINE\_homeo  ------------  Homeobox  HTH\_lambrepressr  SIX  SIX\_SINE\_homeo |
|  | chr9:36500001-37500001 | 9 | PAX5 | Paired box protein Pax-5 (B-cell specific transcription factor) | Paired box protein, N-terminal |
|  | chr3:61800001-62700001 | 9 | FEZL | hypothetical protein FLJ10142 | Znf\_C2H2 |
|  | chr18:23600001-24600001 | 9 | TAF4B | Transcription initiation factor TFIID 105 kDa subunit (TAFII-105) (TAFII105) | TAF4 |
|  | chr8:92200001-93700001 | 9 | CBFA2T1 | Protein CBFA2T1 (MTG8 protein) (ETO protein) (Eigth twenty one protein) (Cyclin D related protein) (Zinc finger MYND domain containing protein 2) | Taf\_hom  Znf\_MYND |
|  | chr8:52400001-53300001 | 9 | ST18 | suppression of tumorigenicity 18 (breast carcinoma) (zinc finger protein) | Znf\_C2HC |
|  | chrX:133800001-134900001 | 9 | ZIC3 | Zinc finger protein ZIC 3 (Zinc finger protein of the cerebellum 3) | Znf\_C2H2 |
|  | chr12:23300001-24500001 | 9 |  |  |  |
|  | chr11:113600001-114600001 | 9 | ZNF145 | Zinc finger protein PLZF (Promyelocytic leukemia zinc finger protein) (Zinc finger protein 145). | Znf\_C2H2  BTB\_POZ |
|  | chr2:103800001-105300001 | 9 | POU3F3 | POU domain, class 3, transcription factor 3 (Brain-specific homeobox/POU domain protein 1) (Brain-1) (Brn-1 protein) | Homeobox  POU\_domain  POU\_homeo |
|  | chr13:34300001-35500001 | 9 |  | NOT ASSIGNED |  |
|  | chr7:41400001-42300001 | 8 | GLI3 | GLI-Kruppel family member GLI3 (Greig cephalopolysyndactyly syndrome) | Znf\_C2H2 |
|  | chr10:50000001-50900001 | 8 | Prrxl1(DRG1) | HOMEOBOX PROTEIN DRG11 HOMOLOG | Homeobox  HTH\_lambrepressr  Homeo\_OAR |
|  | chr17:58900001-60300001 | 8 | TBX2  -----  TBX4 | T-box transcription factor TBX2 (T-box protein 2).  -------  T-box transcription factor TBX4 (T-box protein 4). | P53\_like\_DNA\_bnd  TF\_T-box  ------  P53\_like\_DNA\_bnd  TF\_T-box |
|  | chr5:72100001-73100001 | 8 | FOXD1 | Forkhead box protein D1 (Forkhead-related protein FKHL8) (Forkhead- related transcription factor 4) (FREAC-4). | TF\_Fork\_head |
|  | chr9:118900001-120400001 | 8 | ZNF482  ZNF481  LHX2 |  |  |
|  | chr1:193600001-195100001 | 8 | NR5A2 | Orphan nuclear receptor NR5A2 (Alpha-1-fetoprotein transcription factor) (Hepatocytic transcription factor) | Hormone\_rec\_lig  Stdhrmn\_receptor  Str\_ncl\_receptor  Znf\_C4steroid |
|  | chr1:2200001-3200001 | 7 | PRDM16 | PR-domain zinc finger protein 16 (Transcription factor MEL1) | SET  Znf\_C2H2 |
|  | chr18:29900001-30900001 | 7 |  | NOT ASSIGNED (probably rare transcript) |  |
|  | chr2:118700001-119900001 | 7 | EN1 | Homeobox protein engrailed-1 (Hu-En-1). | Engrailed  Homeobox  HTH\_lambrepressr |
|  | chr12:110900001-111800001 | 7 | CUTL2 | Homeobox protein Cux-2 (Cut-like 2) | Cut\_homeo  Homeobox |
|  | chr6:108000001-108900001 | 7 | NR2E1 | Orphan nuclear receptor NR2E1 (Nuclear receptor TLX) (Tailless homolog) (Tll) (hTll | Hormone\_rec\_lig  Stdhrmn\_receptor  Str\_ncl\_receptor  Znf\_C4steroid |
|  | chr9:102700001-103600001 | 7 | FLJ14960 | Hypothetical protein FLJ14960 | Znf\_C2H2 |
|  | chr3:115000001-115900001 | 7 | ZNF80  -------  ZNF288 | Zinc finger protein 80 (ZNFPT17)  --------  Zinc finger protein 288 (Dendritic-derived BTB/POZ zinc finger protein) | Znf\_C2H2  Znf\_C2H2\_sub  ------  BTB\_POZ  Znf\_C2H2 |
|  | chr6:50400001-51900001 | 7 | TFAP2B | Transcription factor AP-2 beta (AP2-beta) (Activating enhancer-binding protein 2 beta). | TF\_AP2  TF\_AP2\_beta |
|  | chr4:15600001-16600001 | 7 | LDB2 | LIM domain binding 2 | LIM\_bind |
|  | chr7:155700001-156800001 | 7 | HLXB9 | Homeobox protein H9 | Homeobox  Homeodomain\_like |
|  | chr17:1600001-2500001 | 6 | HIC1 | Hypermethylated in cancer 1 | BTB\_POZ  Znf\_C2H2 |
|  | chr1:239300001-240700001 | 6 | ZNF238 |  |  |
|  | chr2:222700001-223600001 | 6 | PAX3 | Paired box protein Pax-3 (HUP2). | Homeobox  Paired\_box  Paired\_homeo |
|  | chr7:12900001-14100001 | 6 | ETV1 | Ets translocation variant 1 (ER81 protein). | ETS  ETS\_PEA3\_N  HSF\_ETS |
|  | chr9:128400001-129200001 | 6 | BARHL1 | BarH-like 1 homeobox protein | Homeobox  HTH\_lambrepressr |
|  | chr17:37200001-38100001 | 6 | NEUROD2 | NeuroD-related factor 2 | HLH\_basic |
|  | chr14:52000001-52900001 | 6 |  | NOT ASSIGNED ( not a cluster?) |  |
|  | chr1:196200001-197100001 | 6 | NR5A2  -------  ZNF281 |  |  |
|  | chr9:8100001-9000001 | 6 |  | NOT ASSIGNED (exons?) |  |
|  | chr14:31100001-32100001 | 6 | NPAS3 | Neuronal PAS domain protein 3 (Neuronal PAS3) (Member of PAS protein 6) (MOP6). |  |
|  | chr8:71500001-72500001 | 6 | EYA1 | Eyes absent homolog 1. |  |
|  | chr20:22000001-22900001 | 5 | FOXA2 | Hepatocyte nuclear factor 3-beta (HNF-3B) (Forkhead box protein A2). | TF\_Fork\_head |
|  | chr1:17700001-18600001 | 5 | PAX7 | Paired box protein Pax-7 (HUP1) | Homeobox  Paired\_box  Paired\_homeo |
|  | chr6:41100001-41900001 | 5 | FOXP4 | forkhead box P4 | TF\_Fork\_head  Wing\_hlx\_DNA\_bnd  Znf\_C2H2 |
|  | chr10:23200001-24100001 | 5 | PTF1A | PANCREAS SPECIFIC TRANSCRIPTION FACTOR, 1A; PANCREAS TRANSCRIPTION FACTOR1 P48 SUBUNIT | HLH\_basic |
|  | chr1:43700001-44600001 | 5 | FLJ40160  ----------  DMAP1 | DNA methyltransferase 1-associated protein 1 (DNMT1-associated protein 1) (DNMAP1) | Znf\_C2H2  ----------  DMAP1  Myb\_DNA\_binding |
|  | chr22:25400001-26200001 | 5 |  | NOT ASSIGNED (close to Meningoma 1) |  |
|  | chr4:1000001-1900001 | 5 | HPX153 | HPX-153 protein | Homeobox  Homeodomain\_like |
|  | chr4:109000001-109900001 | 5 | LEF1 | Lymphoid enhancer binding factor 1 (LEF-1) (T cell-specific transcription factor 1-alpha) (TCF1-alpha) |  |
|  | chr5:121900001-122800001 | 5 | PRDM6 | PR-domain zinc finger protein 6 | SET  Znf\_C2H2 |
|  | chr12:109600001-110500001 | 5 |  | NOT ASSIGNED |  |
|  | chr6:137300001-138200001 | 5 | OLIG3 | oligodendrocyte transcription factor 3 | HLH\_basic  P53\_TF |
|  | chr1:49900001-50700001 | 5 | DMRTA2 | DMRT-like family A2 | DMA  DM\_DNA\_binding |
|  | chr6:6600001-7500001 | 5 | RREB1 | ras responsive element binding protein 1 | Znf\_C2H2 |
|  | chr10:133900001-134800001 | 5 | NKX6-2 | Homeobox protein NKX6-2 | Homeobox |
|  | chr1:25800001-26700001 | 4 | SMARC1 | BRG1-associated factor 250a | ARID  ARM |
|  | chr3:9900001-10800001 | 4 |  | NOT ASSIGNED (probably rare transcript) |  |
|  | chr12:16400001-17300001 | 4 | DAT1 | Neuronal specific transcription factor DAT1 | LIM |
|  | chr15:92600001-93300001 | 4 |  | similar to GLIOMA TUMOR SUPPRESSOR CANDIDATE REGION GENE 2�������������������� PROTEIN (P60) [Homo sapiens] |  |
|  | chr3:129100001-129900001 | 4 | GATA2 | Endothelial transcription factor GATA-2 | Znf\_GATA |
|  | chr16:79700001-80600001 | 4 | MAF | Transcription factor Maf (Proto-oncogene c-maf). | Euk\_transcr\_DNA  TF\_bZIP  TF\_Maf |
|  | chr10:8200001-9100001 | 4 | GATA3 | Trans-acting T-cell specific transcription factor GATA-3 | Znf\_GATA |
|  | chr9:900001-1400001 | 4 | DMRT1  DMRT2  DMRT3 | Doublesex- and mab-3-related transcription factor 1 | DM\_DNA\_binding |
|  | chr4:141100001-142000001 | 4 | MAM3 | mastermind-like 3 (Drosophila) |  |
|  | chr14:72300001-73100001 | 4 | CHX10 | HOMEOBOX PROTEIN CHX10 (CEH-10 HOMEODOMAIN CONTAINING HOMOLOG). | Homeobox |
